# Heterozygous diploid structure of *Amorphotheca resinae* ZN1 contributes efficient biodetoxification on solid pretreated corn stover

**DOI:** 10.1186/s13068-019-1466-z

**Published:** 2019-05-21

**Authors:** Xia Yi, Qiuqiang Gao, Lei Zhang, Xia Wang, Yanqing He, Fengxian Hu, Jian Zhang, Gen Zou, Shihui Yang, Zhihua Zhou, Jie Bao

**Affiliations:** 1 State Key Laboratory of Bioreactor Engineering, East China University of Science and Technology, Shanghai, 200237 China; 2 Jiangxi Provincial Laboratory of Systems Biomedicine, Jiujiang University, 17 Lufeng Road, Jiujiang, 332000 China; 3 CAS Key Laboratory of Synthetic Biology, Institute of Plant Physiology and Ecology, Shanghai Institutes for Biological Sciences, Chinese Academy of Sciences, Shanghai, 200032 China; 4 Hubei Key Laboratory of Industrial Biotechnology, College of Life Sciences, Hubei University, Wuhan, 430062 China

**Keywords:** Biodetoxification, *Amorphotheca resinae* ZN1, Heterozygous diploid, Gene pair, Coordinate expression

## Abstract

**Background:**

Fast, complete, and ultimate removal of inhibitory compounds derived from lignocellulose pretreatment is the prerequisite for efficient production of cellulosic ethanol and biochemicals. Biodetoxification is the most promising method for inhibitor removal by its unique advantages. The biodetoxification mechanisms of a unique diploid fungus responsible for highly efficient biodetoxification in solid-state culture was extensively investigated in the aspects of cellular structure, genome sequencing, transcriptome analysis, and practical biodetoxification.

**Results:**

The inborn heterozygous diploid structure of *A. resinae* ZN1 uniquely contributed to the enhancement of inhibitor tolerance and conversion. The co-expression of gene pairs contributed to the enhancement of the degradation of lignocellulose-derived model inhibitors. The ultimate inhibitors degradation pathways and sugar conservation were elucidated by microbial degradation experimentation as well as the genomic and transcriptomic sequencing analysis.

**Conclusions:**

The finding of the heterozygous diploid structure in *A. resinae* ZN1 on biodetoxification took the first insight into the global overview of biodetoxification mechanism of lignocellulose-derived inhibitors. This study provided a unique and practical biodetoxification biocatalyst of inhibitor compounds for lignocellulose biorefinery processing, as well as the synthetic biology tools on biodetoxification of biorefinery fermenting strains.

**Electronic supplementary material:**

The online version of this article (10.1186/s13068-019-1466-z) contains supplementary material, which is available to authorized users.

## Background

Pretreatment is the central step of biorefinery processing chain to release fermentable sugars from lignocellulose biomass [1–3]. Harsh pretreatment operation causes the generation of various small molecules, including furan aldehydes from over-degradation of pentose and hexose sugars such as furfural and 5-hydroxymethylfurfural (HMF), weak organic acids from acetyl group hydrolysis or aldehyde oxidation such as acetic acid, formic acid, and levulinic acid, as well as phenolic aldehydes from lignin degradation such as 4-hydroxybenzaldehyde (HBA), vanillin, and syringaldehyde [4–7]. The fast and ultimate removal of inhibitors from pretreated lignocellulose biomass avoids their harsh inhibition on cell growth and metabolism of consequent fermenting strains for production of biofuels and bio-based chemicals.

Among various detoxification options, biological degradation of inhibitor compounds by specific microorganisms provides the most promising way for its environment friendly properties [8]. Currently, naturally occurring microorganisms converting inhibitor compounds to less toxic derivatives had been isolated and applied in biorefinery processes [9–17]. However, the overwhelmingly conducted biodetoxification was submerged liquid culture either in pretreatment liquor (a liquid stream generated from pretreatment) or enzymatic hydrolysate (the lignocellulose slurry containing sugars, inhibitors, and lignin residue). Several inherent disadvantages also reduce the feasibility of submerged liquid biodetoxification for practical application. Submerged liquid biodetoxification just incompletely converts low concentrated inhibitors to less toxic intermediates, such as furfural to furfuryl alcohol. When submerged liquid biodetoxification is conducted in pretreatment liquor, a considerable xylose is consumed by biodetoxification strains. In addition, advanced pretreatment technologies generate less liquid waste and even no longer liquid streams [18, 19]. When submerged liquid biodetoxification is conducted in enzymatic hydrolysate, the cellulase enzyme activity is significantly inhibited by the inhibitors before the hydrolysis and the highly concentrated fermentable sugars are massively consumed by the detoxification strains. To reduce the heavy sugar loss of enzymatic hydrolysates, the biodetoxification has to be conducted very quickly and high cell mass for biodetoxification is required as the whole-cell biocatalysts [17].

Direct removal of inhibitors from solid pretreated lignocellulose biomass conserving fermentable sugars is the only feasible option for biodetoxification. A kerosene fungus *Amorphotheca resinae* ZN1 was found biodetoxifies the inhibitors quickly and ultimately from the solid pretreated lignocellulose biomass without fermentable sugar loss and high cell mass requirement. The record of high conversion ethanol [20], chiral lactic acid [21, 22], citric acid [23], and gluconic acid [24] were achieved from the solid-state biodetoxification by *A. resinae* ZN1 [14]. The previous study only concerned the limited understanding of *A. resinae* fungus on hydrocarbon catabolism and glycoside hydrolase [25–28].

In this study, we report an inborn heterozygous diploid structure of *A. resinae* ZN1 and its unique contribution to elevate the competence of inhibitor tolerance and conversion. *A. resinae* ZN1 is able to fulfill the complete degradation according to the predicted ultimate degradation pathways of the model lignocellulose-derived inhibitors. The co-expression of gene pairs significantly further confirmed the enhancement of the degradation of lignocellulose-derived model inhibitors. This is the first observation of the heterozygous diploid structure of biodetoxification strains directly and ultimately degrading inhibitor compounds from solid pretreated lignocellulose biomass. This study provides the new synthetic biology tools for engineering of more effective biodetoxification strains and robust fermenting strains in biorefinery applications.

## Results

### Biodetoxification performance of the newly isolated *A. resinae* ZN1

**Fig. 1:**
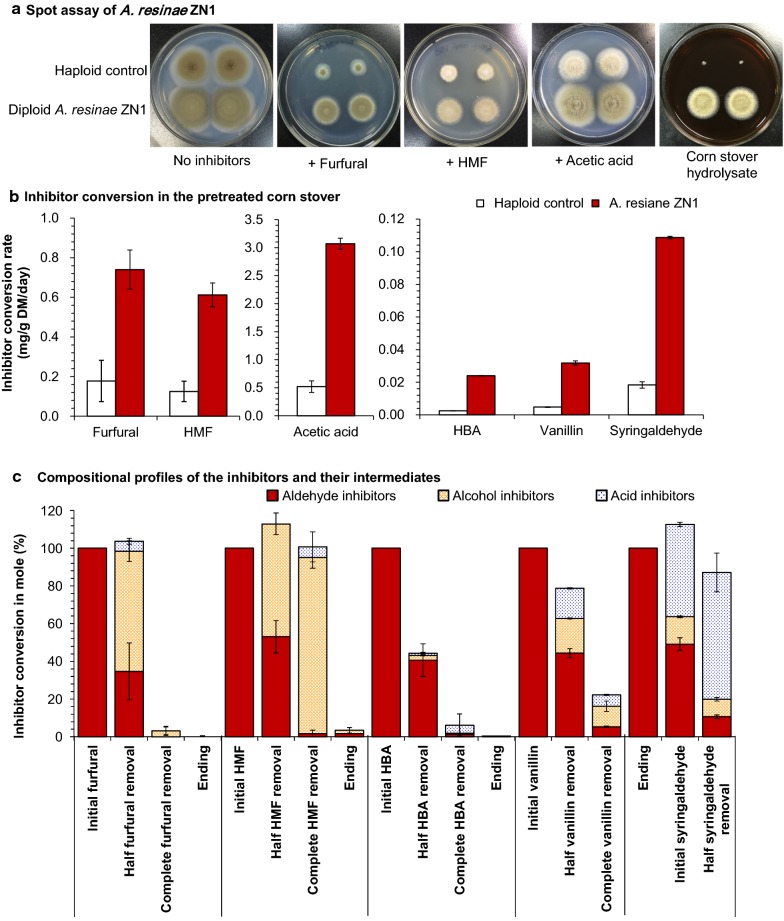
Biodetoxification of lignocellulose-derived inhibitors by *A. resinae* ZN1. **a** Spot assay of *A. resinae* ZN1 and *A. resinae* ATCC 22711 on synthetic medium agar with 5.0-g/L glucose. Conidia were collected and normalized to a final concentration of 1 × 10^8^/mL in sterile water containing 0.05% Tween-80. An equal volume of the solution (0.2 μL) was spotted onto the synthetic medium plates containing various inhibitors (0.5-g/L furfural, 1.5-g/L HMF, and 4.0-g/L acetic acid, respectively) and non-detoxified 15% corn stover hydrolysate (CSH) plate, and then cultured at 28 °C for 6 days (for furfural, HMF, and CSH) or 10 days (for acetic acid). The CSH plate was prepared with 1.5% agar in the non-detoxified 15% CSH. **b** Inhibitor conversion rates of pretreated corn stover under the static conditions by *A. resinae* ZN1 and *A. resinae* ATCC 22711. Detoxification was performed at room temperature (25–28 °C) and pH 5.5. **c** Compositional profiles of intermediates during aldehyde inhibitor conversion. The mole concentration of each inhibitor intermediate accounts for the initial total aldehyde inhibitor mole concentration at different conversion point. “Ending” means the conversion experiment ended. The appropriate inhibitors were added into the liquid synthetic complete medium separately. *A. resinae* ZN1 strain with inoculum 10% (v/v) was cultured at 28 °C and natural pH without shaking. Mean values are presented with error bars representing two standard deviations

The biodetoxification and fermentation performance of the newly isolated fungus *A. resinae* ZN1 were investigated on the model inhibitors derived from lignocellulose by comparing with the haploid fungus *A. resinae* ATCC 22711 as its control (Fig. 1). It was obviously stronger in inhibitor tolerance for *A. resinae* ZN1 with its fast sporulation and mycelium growth on the agar containing furfural, HMF, acetic acid, and corn stover hydrolysate, while the control strain *A. resinae* ATCC 22711 grew slowly (Fig. 1a). It demonstrated that the inhibitor conversion rate of *A. resinae* ZN1 was also higher by three-to-seven fold than that of the control in the practical acid pretreated corn stover solid system (Fig. 1b).

It is generally achieved biodetoxification by converting the more toxic inhibitors to the less ones, but it just achieved the high yield and high titer fermentability after the ultimate degradation of the inhibitors to CO_2_ and water [10, 12, 13, 15, 17]. The HPLC and GC/MS analysis showed that *A. resinae* ZN1 converted the two furan aldehydes (furfural and HMF) and the three phenolic aldehydes (4-hydroxybenzaldehyde, vanillin, and syringaldehyde) to the corresponding alcohols (furfuryl alcohol, HMF alcohol, 4-hydroxybenzyl alcohol, and vanillyl alcohol) and acids (furoic acid, HMF acid, 4-hydroxybenzoic acid, vanillic acid, and syringic acid) before the acids were ultimately assimilated (Fig. 1c).

### Identification of heterozygous diploid structure of *A. resinae* ZN1

The de novo genome assembly with an average coverage of 78× resulted in a 53.4-Mb assembly with GC content of 48.93% and the maximum length scaffold of 1.33 Mb, containing 1014 scaffolds with N50 of 3.75 Mb (Table 1). There were 18,830 coding sequence genes with an average sequence length of 1885 bp. The genome contains 54,079 CDS, 55,111 exons, and 36,281 introns. Genome sequencing data revealed that it was nearly approximately doubled for genome size and protein-coding gene number of *A. resinae* ZN1 (54.47 Mb and 18,830 genes) than that of the haploid *A. resinae* ATCC 22711 (28.63 Mb and 9642 genes).

**Table 1:** General features of the *A. resinae* ZN1 genome

Features	Values
Genome assembly (Mb)	53.4
Total number of scaffold	1014
N50 scaffold length (bp)	375,415
L50 scaffold length (bp)	48
Max scaffold length	1,330,227
GC content (%)	48.93
Number of coding sequence genes	18,830
Average gene length (bp)	1885
Numbers of CDS	54,079
Number of exon	55,111
Number of intron	36,281
Number of genes with intron	15,180

On the other hand, the homology gene family analysis revealed that the gene family number in *A. resinae* ZN1 (8595) was approximately the same with that in *A. resinae* ATCC 22711 (8237), and 8145 gene families were shared by the two *A. resinae* strains. There were two genes locating at different scaffolds for each of the 6794 gene families in *A. resinae* ZN1, but only one gene was for each gene family in *A. resinae* ATCC 22711. Here, we define one gene pair as the two genes in a single gene family in *A. resinae* ZN1. We randomly selected 15 gene pairs (totally 30 genes) as the marker genes from the total 15 gene families for confirmation of gene pair existence. Each of the 15 selected gene families in *A. resinae* ZN1 corresponded to the one of the total 15 scaffolds in *A. resinae* ATCC 22711. It completely agreed with the genomic data for the sequence similarity of the 30 marker genes after being PCR amplified and sequenced from a single spore of *A. resinae* ZN1 (Additional file 1: Figure S1), thus confirming the existence of the gene pairs and two sets of genomes with high homologous similarity in *A. resinae* ZN1. It eliminated the possibility of the two sets of genomes by sexual reproduction in *A. resinae* ZN1 just containing four mating-type genes HMG domain (MAT1-2) (ARZ_8055_T1 and ARZ_2663_T1, ARZ_18448_T1, and ARZ_13604_T1) without alpha-box (MAT1-1) genes.

**Fig. 2:**
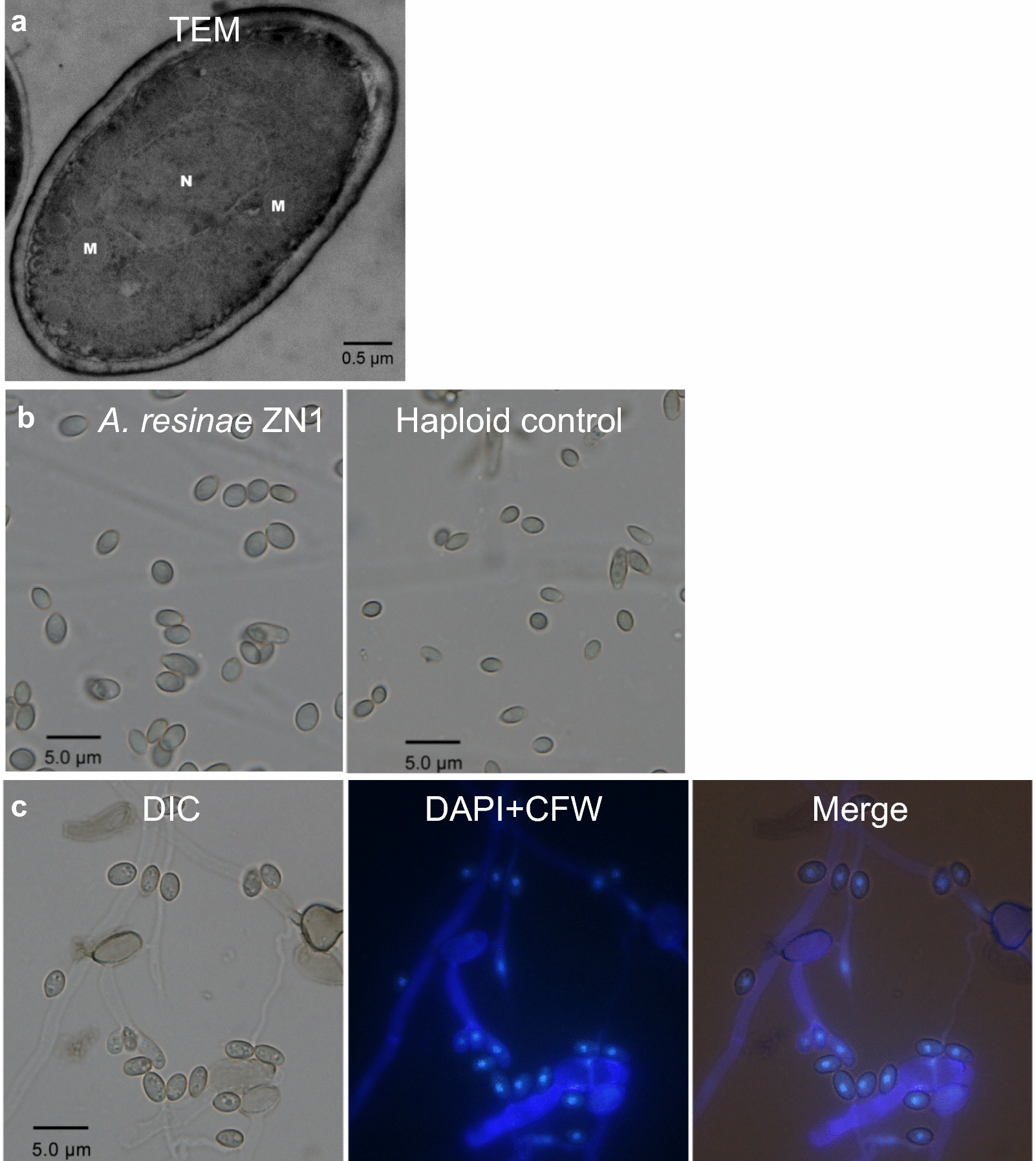
Microscopic images of *A. resinae* ZN1. **a** Transmission electron microscopy (TEM) image showing single nucleus and multiple mitochondria in conidiospore of *A. resinae* ZN1. **b** Conidium images of *A. resinae* ZN1 and the haploid control *A. resinae* ATCC 22711. **c** Fluorescence microscopy images showing one nucleus in each conidium of *A. resinae* ZN1 staining with 4,6-diamidino-2-phenylindole (DAPI) and calcofluor white (CFW), respectively. N and M separately indicated nucleus and mitochondrion, differential interference contrast (DIC) for bright field channel, DAPI + CFW for UV channel, and merge meaning the bright light channel and UV channel

It revealed that each conidium was of just one nucleus but multiple mitochondria by the morphology observation with transmission electron microscopy (TEM) in *A. resinae* ZN1 (Fig. 2a). The nucleus was located in a single conidium by fluorescence microscopy image (Fig. 2c), thus suggesting the existence of a heterozygous diploid structure with the two sets of genomes in one nucleus in *A. resinae* ZN1. The diameter of conidium of *A. resinae* ZN1 was greater than that of the haploid fungus *A. resinae* ATCC 22711 (Fig. 2b), and thus well fits with the morphological property of the general heterozygous diploid fungi [29].

It also valuated genetic stability of the heterozygous diploid *A. resinae* ZN1 by consecutively transferring on PDA agar for 36 days. It showed that there was no change for conidiophore size and the inhibitor conversion rate in *A. resinae* ZN1 (Additional file 2: Figure S2). Genome DNA of the mycelia from the original, 5th, and 11th transfers was isolated as the templates for PCR. It found that 30 marker genes in the 5th and 11th transfers were in accord with that in the original *A. resinae* ZN1 genome by sequencing the PCR product (Table 2; Additional file 1: Figure S1), and thus confirming the highly conserved genetic stability of the heterozygous diploid in *A. resinae* ZN1.

**Table 2:** Selected marker genes in *A. resinae* ZN1

Haploid *A. resinae* ATCC 22711		Diploid *A. resinae* ZN1		Homology similarity	
Scaffold	Gene ID	Scaffold	Gene ID	ORF (%)	Proteins (%)
1	estExt_Genewise1Plus.C_1_t40069	92	*ARZ_14322_T1*	99.86	99.92
		93	***ARZ_14430_T1***	96.18	96.12
2	e_gw1.2.2111.1	6	*ARZ_2135_T1*	100.00	100.00
		28	***ARZ_6929_T1***	94.14	94.79
3	CE64328_29605	46	*ARZ_9744_T1*	100.00	100.00
		86	***ARZ_13904_T1***	94.73	96.09
4	estExt_fgenesh1_pg.C_4_T10012	64	*ARZ_11870_T1*	100.00	100.00
		61	***ARZ_11534_T1***	97.51	97.93
5	estExt_fgenesh1_pm.C_5_T10315	27	*ARZ_6758_T1*	89.07	90.61
		1	***ARZ_138_T1***	90.15	86.99
6	estExt_Genewise1Plus.C_6_T10318	151	*ARZ_17156_T1*	98.05	100.00
		100	***ARZ_14875_T1***	96.97	97.39
7	e_gw1.7.284.1	60	*ARZ_11497_T1*	99.90	100.00
		31	***ARZ_7445_T1***	98.46	98.27
8	fgenesh1_pm.8_#_399	2	*ARZ_581_T1*	99.94	100.00
		105	***ARZ_15233_T1***	97.42	99.24
9	CE116897_14148	55	*ARZ_10881_T1*	100.00	100.00
		7	***ARZ_2411_T1***	97.35	98.40
10	estExt_Genewise1.C_10_T10202	50	*ARZ_10290_T1*	100.00	100.00
		8	***ARZ_2545_T1***	95.76	95.97
11	e_gw1.11.426.1	48	*ARZ_9991_T1*	99.25	99.00
		108	***ARZ_15400_T1***	90.89	90.00
12	e_gw1.12.147.1	53	*ARZ_10682_T1*	99.33	99.32
		30	***ARZ_7271_T1***	96.97	96.96
13	gm1.8671_g	5	*ARZ_1809_T1*	99.19	99.71
		12	***ARZ_3780_T1***	97.46	97.79
14	gm1.8732_g	84	*ARZ_13700_T1*	98.39	99.35
		118	***ARZ_15925_T1***	96.90	98.64
15	e_gw1.15.374.1	69	*ARZ_12452_T1*	99.53	99.39
		37	***ARZ_8414_T1***	97.77	98.17

It selected 15 gene pairs (30 genes) in *A. resinae* ZN1 corresponding to the total 15 scaffolds of *A. resinae* ATCC 22711 with protein-coding genes as marker genes. The one with the higher homology similarity to the corresponding single gene in *A. resinae* ATCC 22711 was defined as the gene pair-I labelled with italic, and the other one was defined as the gene pair-II labelled with bold italic

### Ultimate degradation pathways of inhibitors in *A. resinae* ZN1

**Fig. 3:**
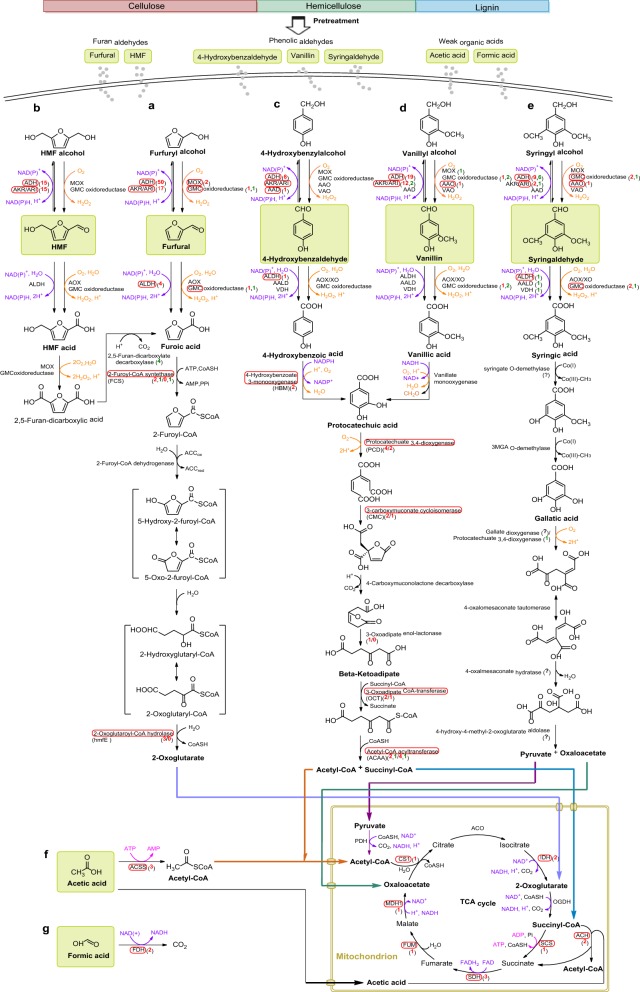
The predicted metabolic pathway of lignocellulose-derived inhibitors. **a**–**g** indicated the predicted degradation pathway of furfural, HMF, 4-hydroxybenzaldehyde, vanillin, syringaldehyde, acetic acid, and formic acid, respectively. The differentially expressed gene pairs were marked with red color. The numbers marked with red and green in bracket separately indicated differentially up-regulated and down-regulated genes during the degradation of the inhibitors. *ACAA* acetyl-CoA acetyltransferase, *ACSS* acetyl-CoA synthetase, *ACO* aconitate hydratase, *ADH* alcohol dehydrogenase, *MOX* alcohol oxidase, *ALDH* aldehyde dehydrogenase, *AKR/ARI* aldo/keto reductase/aldehyde reductase, *AAD* aryl-alcohol dehydrogenase, *AAO* aryl-alcohol oxidase, *CMC* 3-carboxymuconate cycloisomerase, *CS1* citrate synthase (peroxisomal), *FDH* formate dehydrogenase, *FCS* 2-furoyl-CoA synthetase, *FUM* fumarate hydratase, *GMC oxidoreductase* glucose–methanol–choline (Gmc) oxidoreductase, *HBM* 4-hydroxybenzoate 3-monooxygenase, *IDH* isocitrate dehydrogenase, *MDH1* malate dehydrogenase (mitochondrial), *OCT* 3-oxoadipate CoA-transferase, *OGDH* oxoglutarate dehydrogenase complex, *hmfE* 2-oxoglutaroyl-CoA hydrolase, *PCD* protocatechuate 3,4-dioxygenase, *PDH* pyruvate dehydrogenase, *SDH* succinate dehydrogenase, *VAO* vanillyl-alcohol oxidase

According to RNA-Seq transcriptional profiling of *A. resinae* ZN1, it showed that totally 609, 314, 187, 330, 281, 361, and 136 genes were differentially up-regulated during the conversion of furfural, HMF, 4-hydroxybenzaldehyde, syringaldehyde, vanillin, acetic acid, and formic acid, respectively, while 380, 419, 118, 278, 238, 401, and 531 genes were differentially down-regulated, respectively (Additional file 3: Figure S3). Among the differentially expressed genes (DEGs), 291, 68, 30, 168, 123, 151, and 146 genes were significantly differentially expressed during the conversion of the above inhibitors, respectively. It tried to construct the ultimate degradation pathways of the seven model inhibitors in *A. resinae* ZN1 based on the metabolic experimental results, the genome annotation, the transcriptome analysis, and the homologous matching of relevant proteins (Fig. 3).

#### Furan aldehyde inhibitors

The putative pathway of furan degradation was elicited from our RNA-Seq data and the other known species [12]. *A. resinae* ZN1 grew with furfural and/or HMF as the sole carbon source and transformed furfural/HMF to CO_2_ and water by TCA cycle (Fig. 3a, b). There were two parallel pathways to convert furfural to furoic acid catalyzed by oxidoreductases (Fig. 3a). In the first pathway, furfural was reduced to less toxic furfuryl alcohol reversibly oxidized into the low-level furfural by alcohol dehydrogenases (ADH) and aldehyde-ketone reductases/aldehyde reductases (AKR/ARI), and then, the low concentrated furfural was oxidized to furoic acid catalyzed by aldehyde dehydrogenases (ALDH). In the second pathway, furfural or furfuryl alcohol was directly oxidized to furoic acid by free oxygen catalyzed by glucose–methanol–choline (GMC) oxidoreductases, alcohol oxidases (MOX), and aldehyde oxidases (AOX). Furoic acid was assimilated to furoyl-CoA catalyzed by furoyl-CoA ligases and then to 2-oxoglutarate entering TCA cycle catalyzed by furoyl-CoA dehydrogenase and oxoglutaroyl-CoA hydrolase.

HMF conversion referred to two more steps from HMF acid to furoic acid. HMF acid was oxidized to 2,5-furandicarboxylic acid catalyzed by GMC oxidoreductases, MOX, and AOX, and then to furoic acid by 2,5-furandicarboxylate decarboxylase (Fig. 3b). The number of the DEGs responded by furfural was fourfold more than that by HMF. Four genes encoding 2,5-furandicarboxylate decarboxylase were significantly down-regulated under furfural stress, thus explaining why furfural conversion was always prior to HMF conversion with HMF acid accumulates extensively [30, 31].

#### Phenolic aldehyde inhibitors

Similar to furan aldehyde conversion, three phenolic aldehydes, 4-hydroxybenzaldehyde, vanillin, and syringaldehyde separately representing the lignin derivatives of *p*-hydroxyphenyl group (H), guaiacyl group (G), and syringyl group (S), were reduced to the less toxic phenolic alcohols and then oxidized to phenolic acids before finally entering TCA cycle by dehydrogenases (ADH, AKR/ARI, and ALDH), oxidases (MOX), GMC oxidoreductases, aryl-alcohol dehydrogenase (AAD), aryl-aldehyde dehydrogenase (AALD), vanillin dehydrogenase (VDH), aryl-alcohol oxidase (AAO), vanillyl-alcohol oxidase (VAO), and AOX/xanthine oxidase (XO) during phenolic aldehyde conversion to phenolic acids (Fig. 3c–e). 4-Hydroxybenzoic acid and vanillic acid were converted to protocatechuic acid catalyzed by 4-hydroxybenzoate 3-monooxygenases and vanillate monooxygenase, respectively, and then ortho-cleaved by protocatechuate 3,4-dioxygenase entering beta-ketoadipate pathway to generate acetyl-CoA and succinyl-CoA before finally entering TCA cycle (Fig. 3c, d).

Different from 4-hydroxybenzaldehyde and vanillin, genes encoding the enzymes on the protocatechuic acid pathway and beta-ketoadipate pathway were obviously inhibited by syringaldehyde. Therefore, it predicted that syringaldehyde was converted to syringic acid via gallate pathway generating acetyl-CoA and oxaloacetate and then entering TCA cycle (Fig. 3e).

Two and six genes encoding laccase were also significantly differentially expressed during the conversion of vanillin and syringaldehyde, respectively, thus suggesting that the multi-copper phenol oxidase enzyme played a role on the oxidation of phenolic aldehydes by catalyzing ring cleavage.

#### Weak organic acid inhibitors

Acetic acid was first converted to acetyl-CoA by acetyl-CoA synthetases (ACSS) (in cytoplasm) and acetyl-CoA hydrolases (ACH) (in mitochondria) and then entered the TCA cycle with the related genes significantly up-regulated (Fig. 3f). Usually, the glyoxylate cycle was an alternative pathway for acetyl-CoA assimilation, but specific genes encoding isocitrate lyase (ICL) and malate synthase (MS), as well as malate dehydrogenase 2 (MDH2) and citrate synthase 2 (CS2) were not significantly expressed under acetic acid stress, thus suggesting that the glyoxylate cycle was not the bypass of acetic acid metabolism. Formic acid is directly converted to CO_2_ with formate dehydrogenases (FDH) encoding gene significantly up-regulated (Fig. 3g).

**Fig. 4:**
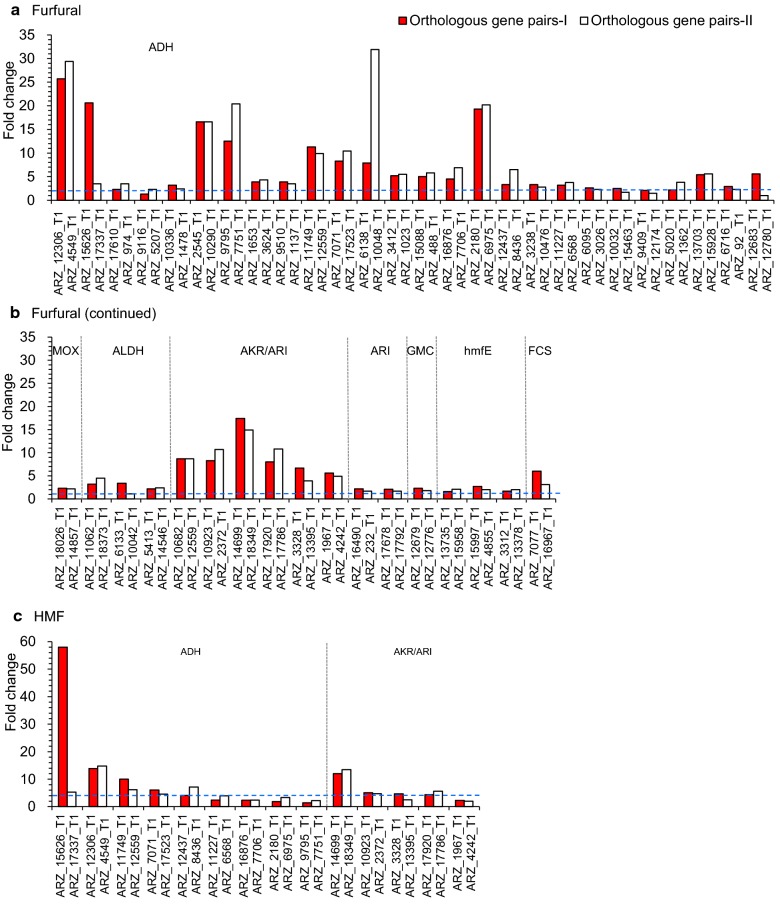

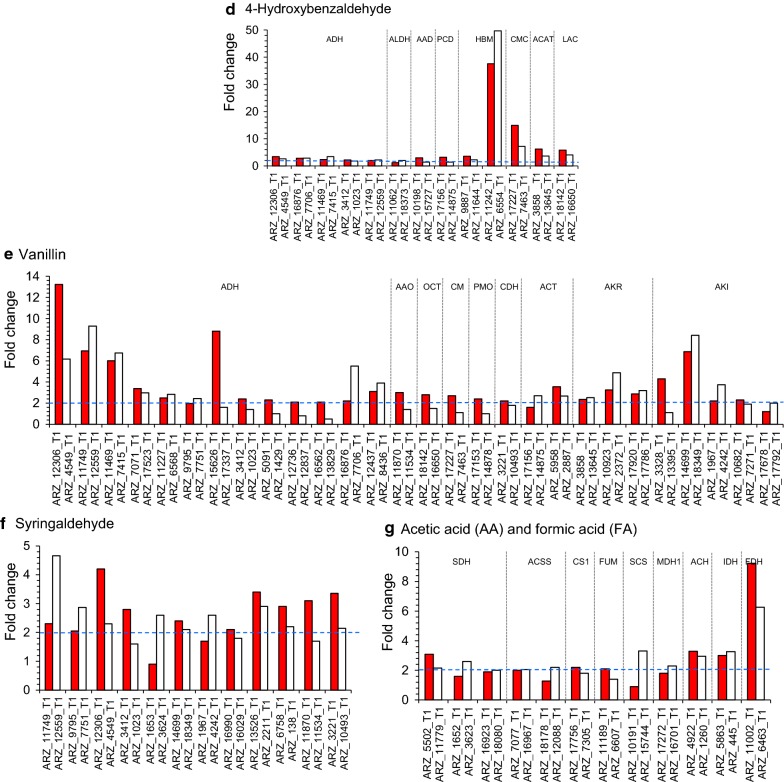
Comparison of the significantly up-regulated expression of orthologous gene pairs in the biodegradation pathway under various inhibitor conditions. **a**-**b** Furfural. **c** HMF. **d** 4-Hydroxybenzaldehyde (4-HBA). **e** Vanillin. **f** Syringaldehyde. **g** Acetic acid (AA) and formic acid (FA). The orthologous gene pairs with each two or at least one of orthologous genes significantly differentially up-regulated were selected and ranked from high to low by fold change under each inhibitor condition

It also investigated the orthologous gene pairs involving with the ultimate degradation pathways of lignocellulose-derived model inhibitors. Figure 4 is the differentially up-regulated expression of the orthologous gene pairs responsible for inhibitor degradation. Totally, 112 gene pairs, including 43 for furfural, 14 for HMF, 13 for 4-hydroxybenzaldehyde, 28 for vanillin, 12 for syringaldehyde, and 12 for acetic acid and formic acid, were closely related with the degradation of the above inhibitors. At least one or two genes together in each orthologous gene pair were differentially up-regulated, especially for the gene pairs encoding alcohol dehydrogenase (ADH) and aldehyde-ketone reductases/aldehyde reductases (AKR/ARI) differentially expressed and enriched during the conversion of five aldehyde inhibitors. The synergistic and complementary expression of the gene pairs assured the minimum enzyme activities and maintained potentials for maximum activities in each conversion step.

### Sugars conservation during biodetoxification in *A. resinae* ZN1

It is important to maximize the conservation of fermentable sugars during biodetoxification for the achievement of a high yield of target products in the consequent fermentation step. It showed that inhibitor degradation prior to sugars consumption in *A. resinae* ZN1 contributed to the maximum sugar conservation in biodetoxification (Table 3). Ultimate degradation of the most toxic furfural, HMF and highly concentrated acetic acid accompanied with the consumption of only less than 1.6% of the total sugar in the feedstock. The bioconversion of phenolic aldehydes (4-hydroxybenzaldehyde, vanillin, and syringaldehyde) started when furfural was completely removed with less than 6.1% of the total sugar consumption.

**Table 3:** Inhibitor conversion and sugar consumption by *A. resinae* ZN1 on solid corn stover after acid pretreatment

	Furfural	HMF	Acetic acid	HBA	Vanillin	Syringaldehyde
Inhibitor concentration (mg/g DM)	5.52 ± 0.22	2.26 ± 0.04	15.91 ± 2.05	0.19 ± 0.02	2.41 ± 0.09	1.12 ± 0.36
Complete conversion time (h)	36	36	36	56^a^	72^a^	72^a^
Glucose consumption (mg/g DM)	5.49 ± 1.09	5.49 ± 1.09	5.49 ± 1.09	9.64 ± 0.20	10.08 ± 0.44	10.08 ± 0.44
Xylose consumption (mg/g DM)	3.77 ± 1.75	3.77 ± 1.75	3.77 ± 1.75	18.97 ± 1.09	25.92 ± 6.92	25.92 ± 6.92
Total sugar loss (%)	1.56 ± 0.48	1.56 ± 0.48	1.56 ± 0.48	4.83% ± 0.15	6.08% ± 1.24	6.08% ± 1.24

Condition: 5-L bioreactor, 28 °C, pH 5.5, aeration rate of 1.33 vvm (defined as the air volumetric flowrate in liter per minute to the corn stover feedstock volume in liter). Total sugar loss (%) was calculated for the sum of consumed glucose and xylose divided by the total glucose and xylose contained in the solid pretreated corn stover. Six model inhibitors without formic acid were analyzed due to the very less formic acid on the real solid feedstocks after acid pretreatment. All experiments were carried out in duplicate. Error was calculated as standard deviation

^a^The conversion rate about 90% for the three phenolic aldehydes achieved at this time point

**Fig. 5:**
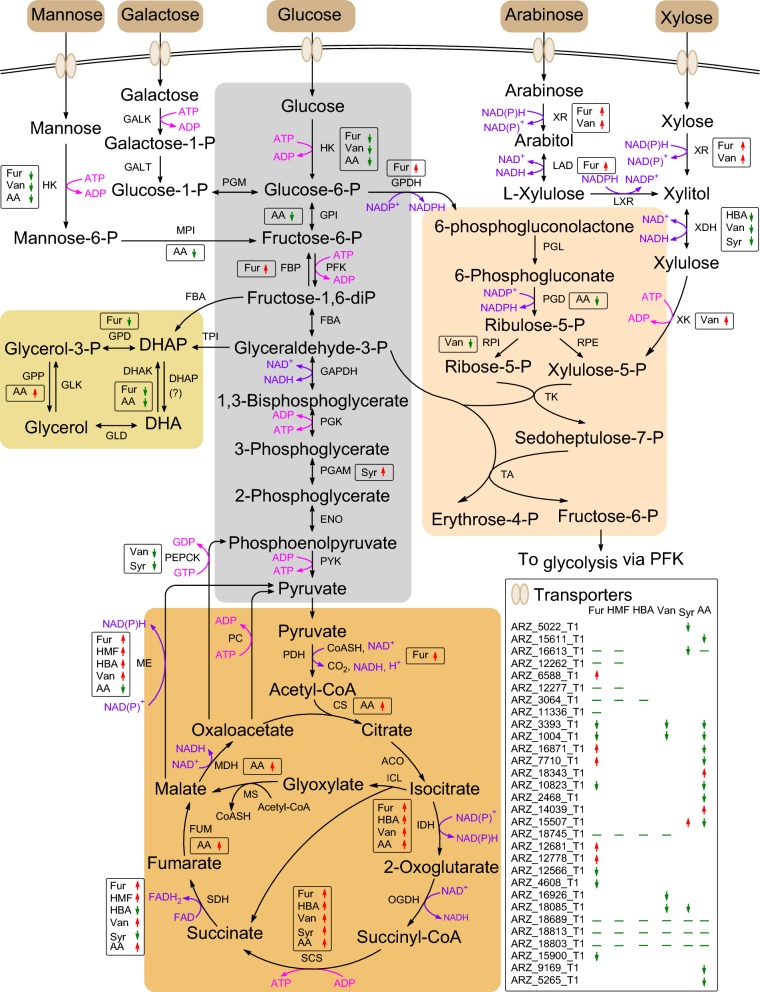
Reconstruction of central and lignocellulose contained sugars metabolism of *A. resinae* ZN1. Arrow marked with red and green separately indicated the up-regulated and down-regulated expressed genes under aldehyde inhibitor degradation. *AA* acetic acid, *ACO* aconitate hydratase, *ADP* adenosine diphosphate, *ATP* adenosine triphosphate, *CS* citrate synthase, *DHAK* glycerol dehydrogenase K, *DHAP* glycerol dehydrogenase P, *ENO* enolase, *FBA* fructose-bisphosphate aldolase, *FBP* fructose-1,6-bisphosphatase, *FUM* fumarase, *Fur* furfural, *GALK* galactokinase, *GALT* galactose-1-phosphate uridylyltransferase, *GAPDH* glyceraldehyde-3-phosphate dehydrogenase, *GDP* guanosine diphosphate, *GLD* glucose dehydrogenase, *GLK* glucokinase, *GPD* glycerol-3-phosphate dehydrogenase, *GPI* glucose-6-phosphate isomerase, *GPP* glycerol 3-phosphatase, *GTP* guanosine triphosphate, *HBA* 4-hydroxybenzaldehyde, *HK* hexokinase, *IDH* isocitrate dehydrogenase, *LAD* l-arabitol dehydrogenase, *LXR* l-xylulose reductase, *MDH* malate dehydrogenase, *ME* malic enzyme, *MPI* mannose phosphate isomerase, *OGDH* oxoglutarate dehydrogenase, *PC* pyruvate carboxylase, *PDH* pyruvate dehydrogenase, *PEPCK* phosphoenolpyruvate carboxykinase, *PFK* phosphofructokinase, *PGAM* phosphoglycerate mutase, *PGK* phosphoglycerate kinase, *PGD* phosphogluconate dehydrogenase, *PGL* 6-phosphogluconolactonase, *PGM* phosphoglucose mutase, *PYK* pyruvate kinase, *RPE* ribulose-5-phosphate-3-epimerase, *RPI* ribose-5-phosphate isomerase, *SCS* succinyl coenzyme A synthetase, *SDH* succinate dehydrogenase, *Syr* syringaldehyde, *TA* transaldolase, *TK* transketolase, *Van* vanillin, *XDH* xylitol dehydrogenase, *XK* xylose kinase, *XR* xylose reductase

Transcriptome analysis revealed that the genes involving in sugar metabolism were differentially down-regulated during the conversion of the inhibitors in *A. resinae* ZN1 (Fig. 5; Additional file 4: Dataset S1). Except for arabinose and galactose transporter encoding genes inhibited, majority of the sugar transporter encoding genes were differentially down-regulated during the inhibitor degradation. Hexokinase (HK) genes in the first and the rate-limiting step were highly inhibited by furfural, vanillin, and acetic acid in the glycolysis pathway. GPI encoding glucose-6-phosphate isomerase in the second step was also differentially inhibited by acetic acid. PYK encoding pyruvate kinase, the rate-limiting enzyme, was relatively down-regulated by the above three inhibitors. FBP encoding fructose bisphosphatase in gluconeogenesis was differentially induced by furfural, and, thus, indicated that the furfural inhibition led to the low sugar level in the cell. In addition, phosphoenolpyruvate carboxykinase (PEPCK) genes were inhibited by vanillin and syringaldehyde. Two genes encoding phosphogluconate dehydrogenase (PGD) and ribose 5-phosphate isomerase (RPI) in pentose phosphate pathway (PPP) were differentially down-regulated during acetic acid and vanillin conversion. Three genes encoding glycerol 3-phosphate dehydrogenase (GPD) and dihydroxyacetone kinases (DHAK) in glycerol metabolism were differentially down-regulated by furfural and acetic acid. The xylitol dehydrogenase (XDH) and mannose-6-phosphate isomerase (MPI) genes were also differentially down-regulated by three phenolic aldehydes and acetic acid, respectively. It prevented the fast sugar consumption during biodetoxification by the strong inhibition on the sugar transport and central metabolism.

No corresponding information was for the genes encoding endoglucanase (EG) and cellobiohydrolase (CBH) by KEGG annotation in *A. resinae* ZN1 genome. The CAZy annotation showed that four genes (ARZ_13704_T1, ARZ_15929_T1, ARZ_17383_T1, and ARZ_17659_T1) belonged to glycoside hydrolase family 5 and two genes (ARZ_8331_T1 and ARZ_10420_T1) belonged to auxiliary activity family 9, but these putative glycoside hydrolase genes were either down-regulated, or regularly expressed, or below the detection limitation of RNA-seq reads at the transcriptional level during the inhibitors conversion. *A. resinae* ZN1 was unable to hydrolyze cellulose to glucose during biodetoxification supported by the absence or silence of the cellulase encoding genes, and thus was in consistent with the findings of no cellulose hydrolysis and no cell growth on cellulose substrate by *A. resinae* ZN1 [14].

Additional file 5: Figure S4 was the regulation of the two genes locating one orthologous gene pair in central metabolism during the degradation of the various inhibitors. Gene pairs encoding glucose transporter were inhibited by furfural, vanillin, syringaldehyde, acetic acid, and formic acid, and, thus, further elucidated the degradation of the model inhibitors prior to fermentable sugar consumption in *A. resinae* ZN1. All the five aldehyde inhibitors inhibited citrate synthase encoding gene (Fig. 5; Additional file 4: Dataset S1) and the major genes involving in TCA cycle leading to the inhibition of sugar consumption and ATP production. The orthologous gene pairs were up-regulated for IDH, MDH1, ME, OGDH, and PDH during furfural degradation, GAPDH, GPDH, ME, and PGD during HMF degradation, IDH, ME, OGDH, and PDH during 4-hydroxybenzaldehyde degradation, IDH, MDH1, ME, and OGDH during vanillin degradation, IDH, GPDH, ME, and PGD during syringaldehyde degradation, and IDH, MDH1, OGDH, SCS, and SDH during acetic acid degradation. It was suggested that the coordinate up-regulated expression of the gene pairs used for cofactor regeneration contributed to the supply of sufficient NAD(P)H for aldehyde inhibitors and acetic acid degradation [32–36]. The up-regulated expression of orthologous gene pairs facilitated acetic acid assimilation by elevating ATP production and down-regulating ATP consumption.

It indicated that the orthologous gene pairs in the heterozygous diploid *A. resinae* ZN1 efficiently compensated the rate-limiting step of inhibitor conversion by the improvement of central carbon metabolism, cofactor production, and ATP consumption.

## Discussion

It demonstrated that *A. resinae* ZN1 was superior to the most of the reported biodetoxification strains, such as *Trichoderma reesei* RUT-30 [9], *Coniochaeta ligniaria* NRRL30616 [10], *Ureibacillus thermosphaericus* [11], *Cupriavidus basilensis* HMF14 [13], *Issatchenkia occidentalis* CCTCC M 206097 [15], *Aspergillus nidulans* FLZ10 [16], and *Enterobacter* sp. FDS8 [17] as follows: (1) biodetoxification by *A. resinae* ZN1 was conducted on the solid pretreated lignocellulose feedstock without freshwater usage and wastewater generation and with much higher concentrated inhibitors detoxified (two orders of magnitude greater than the reported submerged liquid biodetoxification). (2) All the model inhibitors, such as furan aldehydes (furfural and HMF), phenolic compounds (4-hydroxybenzaldehyde, vanillin, and syringaldehydes) and weak acids (acetic acid and formic acid), were ultimately degraded without the intermediates accumulating and with negligible fermentable sugar consuming. (3) Biodetoxification by *A. resinae* ZN1 was quickly performed on a solid-state fermentation in a fermentative way with low cell inoculation and filamentous fungus or spores grow rather than a whole-cell biocatalysis way at extremely high cell mass requirement.

This study discovered and confirmed the inborn heterozygous diploid structure in *A. resinae* ZN1. Most importantly, it identified that the heterozygous diploid structure of genetic stability without haploid differentiation during its natural inhabitant environment and biodetoxification processes was the inherent and indispensable property for ultimate biodetoxification of lignocellulose-derived inhibitor compounds. Heterozygous diploid fungi were superior to the haploids on cell growth and metabolism diversity [29], but the frequency of spontaneous heterozygous diploid formation was very low in nature (approximately 10^−7^–10^−5^) [37]. Here, we show that the heterozygous diploid *A. resinae* ZN1 was significantly higher in inhibitor tolerance and inhibitor conversion rate compared with its haploid control *A. resinae* ATCC 22711. This property is significantly different from the incomplete conversion to only alcohols or acids by the reported biodetoxification microorganisms [11, 15, 17]. The heterozygous diploid structure of *A. resinae* ZN1 made it more flexible and more powerful on relieving the harsh inhibitor tolerance than the haploid strain.

It was one of the major driving forces on the powerful biodetoxification for the heterozygous diploid *A. resinae* ZN1 that the two genes in each orthologous gene pair were coordinately and differentially up-regulated on the inhibitor degradation pathways and cofactor regeneration (Fig. 4; Additional file 5: Figure S4). The similar regulation pattern applied on the sugar metabolisms in the opposite ways by coordinately down-regulating the expression of the genes responsible for sugar consumption. This fine regulation guaranteed the effective biodetoxification of *A. resinae* ZN1 by complementing on eliminating rate-limiting steps and contributing to the inhibitor degradation prior to sugar consumption. It predicted that *A. resinae* ZN1 was responsible for inhibitor degradation, and selection pressure of inhibitory stress contributed to the formation of gene pairs. Thus, agreed the amelioration of the deleterious effects of toxic intermediate compounds was a metabolic phenotype favored by the sufficient selection pressure in fungi resulting in the formation of gene pairs representing signatures of selection for the protection from toxic metabolics [38].

ATP was mainly required for acetic acid assimilation as well as protons and anions’ pump-out [25, 26]. The electron transport chain (GO: 0022900) and oxidative phosphorylation (GO: 0006119) were significantly enriched during acetic acid stress (Additional file 6: Figure S5), thus, suggesting the accelerated ATP generation on acetic acid conversion. On the contrary, ATP-citrate lyase (ACLY), hexokinase (HK), phosphofructokinase (PFK), phosphoenolpyruvate carboxykinase (PEPCK), and pyruvate carboxylase (PC) involving in ATP were differentially down-regulated under the stress of acetic acid (Additional file 7: Table S1). Therefore, the genes relating to ATP generation and consumption were separately up-regulated and down-regulated ensuring the sufficient supply of ATP for the fast detoxification of acetic acid.

The finding of the heterozygous diploid *A. resinae* ZN1 on biodetoxification took the first insight into the global overview of biodetoxification mechanism of lignocellulose-derived inhibitors. This study provided the new selection criteria for more powerful biodetoxification strain, the valuable thoughts for microbial physiology, and the useful synthetic biology tools for the enhancement of inhibitor robust in the lignocellulose biorefinery processes.

## Conclusions

It found that an inborn heterozygous diploid structure of *A. resinae* ZN1 uniquely contributed to the enhancement of inhibitor tolerance and conversion. *A. resinae* ZN1 could achieve ultimate degradation according to the predicted degradation pathways of the model lignocellulose-derived inhibitors depending genomic and transcriptomic sequencing. The co-expression of gene pairs contributed to the enhancement of the degradation of lignocellulose-derived model inhibitors. The finding of the heterozygous diploid *A. resinae* ZN1 on biodetoxification took the first insight into the global overview of biodetoxification mechanism of lignocellulose-derived inhibitors. This study provided the new selection criteria for unique biodetoxification strain, the valuable thoughts for microbial physiology, and a potential synthetic biology tool to strengthen the inhibitor robustness.

## Methods

### Enzyme and reagents

Corn stover (CS) was harvested from Dancheng, Henan, China. CS was washed and precipitated to remove the field dirt, sands, metal pieces, and other impurities, and then air-dried to a constant weight. The clean CS was ground coarsely using a beater pulverizer and screened through a mesh with the circle diameter of 10 mm. The raw CS contained 36.2% of cellulose and 19.8% of xylan determined by two-step acid hydrolysis method [39]. The cellulase Youtell #6 was purchased from Hunan Youtell Biochemical Co., Yueyang, Hunan, China. The filter paper activity of 63 FPU and the cellobiase activity of 102 CBU per gram of the enzyme were determined according to the methods [40, 41], respectively.

Yeast extract was purchased from Oxiod, Basingstoke, Hampshire, UK. Furfural and 5-hydroxymethylfurfural (HMF) were from J&K Scientific, Beijing, China. 4-Hydroxybenzaldehyde and vanillin were separately from Sangon Biotech and Aladdin Reagents, Shanghai, China. Syringaldehyde was from Alfa Aesar, Heysham, UK. Acetic acid was from Sinopharm Chemical Reagent, Shanghai, China. All other chemicals were purchased from Lingfeng Chemical Reagent Co., Shanghai, China.

### Strains and culture

*Amorphotheca resinae* ZN1 stored at China General Microbiological Culture Collection (CGMCC), Beijing, China, with the registration number of CGMCC 7452 and *A. resinae* ATCC 22711 purchased from American Type Culture Collection (ATCC), Manassas, VA, were cultured on potato–dextrose–agar (PDA) agar medium-containing 200.0 g/L of potato extract juice, 20.0 g/L of glucose, and 15.0 g/L of agar at 28 °C for sporulation [14, 31].

For spot assay, conidia were collected and normalized to a final concentration of 1 × 10^8^/mL in sterile saline water containing 0.05% Tween-80. An 0.2-μL volume of the solution was spotted on the synthetic medium (1.0 g/L of yeast extract, 2.0 g/L of KH_2_PO_4_, 1.0 g/L of (NH_4_)_2_SO_4_, 1.0 g/L of MgSO_4_·7H_2_O, 0.5 g/L of CaCl_2_, and 5.0 g/L of glucose) plates amended with various inhibitors (0.5-g/L furfural, 1.5-g/L HMF, and 4.0-g/L acetic acid, respectively) and non-detoxified 15% corn stover hydrolysate (CSH) plate, and then cultured at 28 °C for 6 days (for furfural, HMF, and CSH) or 10 days (for acetic acid) without shaking. The CSH plate was prepared with 1.5% agar in the non-detoxified 15% CSH.

For biodetoxification, the pretreated corn stover was carried out in a 15-L container at 28 °C. *A. resinae* ZN1 seeds were cultured at 28 °C for 7 days on the pretreated corn stover by inoculation of spores from PDA slant. 10% of seed solids was inoculated onto the newly pretreated corn stover and cultured at 28 °C and pH 5.5 under static condition without shaking for 3 days. The detoxified corn stover was disk-milled before use.

For successive transfer assay of *A. resinae* ZN1, 100 μL conidial suspension from one colony with a final density of 1 × 10^2^ mL^−1^ was prepared and evenly smeared on PDA plate. The single colony was selected after 3–4 days at 28 °C for the next transfer and also used to isolate genomic DNA from the original, 5th and 11th transfers using the FastPrep-24 (MP Biomedicals, Santa Ana, CA) from 30 mL mycelia culture on PDA medium and the mycelia.

For RNA-Seq, a PDA slant of *A. resinae* ZN1 was inoculated in PDA medium at aerobic conditions separately amended with 1.0-g/L (furfural and HMF) and 0.1-g/L phenolic aldehydes (4-hydroxybenzaldehyde, syringaldehyde, and vanillin) as the sample groups at 28 °C without shaking for 12 h, and the control groups were without aldehyde inhibitors. After collection, the samples freshly isolated were immediately used for total RNA extraction and RNA-Seq. The control and treatment groups were in duplicate.

### Single spore isolation and identification

A single spore strain of *A. resinae* ZN1 was obtained according to the protocols with a slight modification [42]. The pretreated corn stover solids that *A. resinae* ZN1 originally grew were used as the complex materials after being autoclaved twice and were placed on the agar surface aseptically. It picked up spores of mycelia directly from the substrate using the SporePlay dissection microscope equipped with a 50 μm in diameter dissection needle (Singer Instruments, Somerset, UK). To provide a spore suspension, the spores were made into a spore suspension placed in sterilized water and agitated. The prepared homogenous spore suspension was then transferred onto the surface of the water agar plate marked 16 squares on the bottom of the water agar plate. The uncontaminated germinated spores were transferred and distributed evenly onto the PDA plate at 28 °C for 3–4 days. The pure culture stored at 4 °C or in liquid nitrogen was used to perform further genome sequencing.

Identification of heterozygous diploid of *A. resinae* ZN1 was used a Hitachi H-7650 TEM at 80 kV (Hitachi, Kyoto, Japan). The conidia were collected from the culture of *A. resinae* ZN1 on PDA plate at 28 °C according to the protocols [43].

Fluorescence microscopy observation was carried out on the conidia collected after being cultured on PDA plate for 4 days and washed with PBS for three times. Spores were stained with 4,6-diamidino-2-phenylindole (DAPI) (Sigma-Aldrich, St. Louis, MO) with the final concentration of 10 μg/mL for 3 min in dark, washed and then stained with 0.001% calcofluor white (Sigma-Aldrich, St. Louis, MO) for 2 min in dark, washed and finally viewed with a OLYMPUS BX51 fluorescence microscope (Olympus, Tokyo, Japan) with a 100× magnification on the UV channel and the bright light channel.

### Pretreatment, enzymatic hydrolysis of corn stover, and solid-state biodetoxification

The dry acid pretreatment was carried out in a helical ribbon impeller-driven reactor at 175 °C for 5 min at a solid-to-liquid ratio of 2:1 (w/w) with 2.5 g of sulfuric acid per 100 g of dry corn stover (2.5% acid usage) according to the methods [44, 45]. The pretreated CS was maintained with solid state at about 45% solid content and contained 40.1% of cellulose, 3.1% of xylan, as well as 13.5 mg of glucose and 97.8 mg of xylose per gram of dry solid matter according to the NREL LAP protocol [39].

Corn stover hydrolysate was prepared by enzymatically hydrolysis of the pretreated corn stover (without detoxification) at 15% (w/w) of solids loading and of 10 mg cellulase protein/g cellulose for 48 h at 50 °C to give the composition of 50.4 g/L of glucose, 23.6 g/L of xylose, 0.21 g/L of furfural, 0.31 g/L of HMF, and 4.43 g/L of acetic acid. Biodetoxification was conducted in the sealed plastic boxes. Two hundred grams of the pretreated CS was neutralized by 20% (w/w) Ca(OH)_2_ slurry to pH 5.5, then inoculated with 1 × 10^8^ spores of *A. resinae* ZN1 or *A. resinae* ATCC 22711, and cultured at room temperature (23–28 °C) without nutrient addition. The mixture of inoculum and pretreated CS occupied 1/4 volume of the box. 5.0 g samples were withdrawn periodically and rinsed with 50 g water then shaken at 30 °C for 1 h to obtain the supernatant for analyzing inhibitors on HPLC.

### Genomic and transcriptomic sequencing

To mine the molecular mechanism of heterozygous diploid in *A. resinae* ZN1, it performed genome sequencing and RNA-Seq. Genomic DNA, extracted by cetyltrimethylammoniumbromide (CTAB) method [46], was used to perform genome sequencing and the biomarker amplification of gene family in *A. resinae* ZN1. The primers of gene families used in this study were listed in Additional file 7: Table S1. Two genomic DNA libraries of *A. resinae* ZN1 with the inserted sizes of 0.5 and 6 kb were constructed and paired-end sequenced using the Illumina HiSeq 2000 system performed by BGI-Shenzhen, China. Cleaning step was carried out using FastQC and PRINSEQ to read quality control and preprocessing before assembly [47, 48]. Approximately 4 Gb of high-quality clean sequences were obtained after filtering and correction of the low-quality, PCR-duplicated, and adapter-contained sequences from the raw data and assembled using the SOAPdenovo (v1.05) software [49]. The genome assembly of *A. resinae* ZN1 was annotated using the Program to Assemble Spliced Alignments (PASA) pipeline (v20130907) [50]. Before gene finding, repeat sequences were predicted by RepeatMasker (Repbase database) [51] and RepeatProteinMasker (RepeatMasker transposon protein database) [52]. De novo repeats were predicted with RepeatModeler [53]. PASA was employed to generate the training sets for Augustus (v2.5.5) [54] and SNAP (v20130216) [55] with the trinity assembly result. Gene models were predicted independently with a set of gene finders: Augustus, GeneMark-ES (v2.3e) [56], and SNAP. The PASA assemblies (including the polyadenylation sites) were used as the hints for Augustus gene prediction. The 18,955 consolidated consensus gene models for each locus were produced by information-based source-weighted integration using EvidenceModeler (EVM, v20120625) [57]. Six RNA-seq data sets from furfural and HMF treatments were mapped to *A. resinae* ZN1 genome sequence using the recommended protocol (the two-step alignments for Ion Proton™ sequencer RNA-seq analysis, http://ioncommunity.lifetechnologies.com/docs/DOC-8434). TopHat2 (v2.0.9) [58] and Bowtie2 (v2.2.1) [59] were employed as aligner in the workflow and the EVM gene models were used as reference transcripts. Finally, the genome-guided trinity assembly was performed using the Ion Proton RNA-seq alignment. Genome-guided trinity assemblies and de novo RNA-seq trinity assemblies were incorporated into PASA pipeline to build a comprehensive transcriptome database. Annotation updates for 18,955 EVM gene models were performed to generate the final gene models, and totally, 18,830 unique gene models were generated through the gene prediction pipeline. In addition to protein-coding genes, tRNAs were predicted using tRNAscan-SE (v1.21) [60]. The software QUAST was used to obtain the statistical data such as assembly size [61]. For gene functional annotation, BLASTp against highly curated databases, such as SwissProt, KEGG (v58), STRING (v9.1), and TCDB, were performed to assign general protein function profiles. The predicted proteins were annotated with KOG classification using the STRING database.

Total RNA was extracted by Trizol Reagent kit (Invitrogen, Carlsbad, CA, USA) following the manufacturer’s instructions. RNA-Seq was performed by NovelBio Bio-Pharm Technology Co., Ltd, Shanghai, China. RNA was purified by NucleoSpin RNA clean-up kit (Macherey–Nagel, Düren, Germany). The quality of RNA was checked by Bioanalyzer 2200 (Aligent Technologies, Santa Clara, CA, USA). RNA samples were kept at − 80 °C. The cDNA libraries, prepared using Ion Total RNA-Seq Kit v2.0 (Life Technologies), were used to work for the Proton Sequencing process. The raw sequence reads were trimmed for low-quality bases and adapter sequences, and 69.75 Gb clean data were obtained. Sixteen transcriptome samples about 19.21 Gb clean data from furfural and HMF treatments were used to de novo assemble by trinity software with strand-specific (v20140413) for annotation [62]. MapSplice software was used for RNA-seq mapping [63]. The gene expression level was normalized to Reads Per Kilobases per Million mapped Reads (RPKM). Based on the counts achieved by HTSeq from the only unique mapped reads, the DESeq algorithm was used to identify and screen the differentially expressed genes (DEGs) for the control and experiment groups [64, 65]. In addition, a fold change of 2.0 was set as the criteria for DEGs, and both a threshold of 0.05 for false discovery rate (FDR) used to control the error rate and a fold change of 2.0 were for significant DEGs. Gene ontology (GO) enrichment analysis was used GOseq (v1.18) based on Wallenius’ non-central hyper-geometric distribution and the gene length to estimate the parameters to finally make the enrichment for the functional classification.

It also carried out orthologous group analysis. A total of 172,600 protein-coding genes from 15 fungal genomes, such as *A. resinae* ZN1, *A. resinae* ATCC 22711, *Aspergillus niger* CBS 513.88, *Aspergillus oryzae* RIB40, *A. nidulans* FGSC A4, *Penicillium chrysogenum* Wisconsin 54–1255, *Trichoderma reesei* QM6a, *Neurospora crassa* OR74A, *Talaromyces marneffei* ATCC 18224, *Saccharomyces cerevisiae* S288C, *Yarrowia lipolytica* CLIB122, *Botryotinia fuckeliana* B05.10, *Magnaporthe oryzae* 70-15, *Phanerochaete chrysosporium* RP-78, and *Fusarium graminearum* PH-1, were performed an all-against-all pairwise BLASTp similarity search, and orthologous group was clustered using OrthoMCL (v2.0) package with *E*-value cut-off of 1*E*−5 and percentage match cut-off of 50 [66]. The procedure resulted in 15,795 gene orthologous groups which at least contain two members. The gene orthologous groups were annotated using PFAM domain database.

### qRT-PCR

It used quantitative real-time polymerase chain reaction (qRT-PCR) to validate RNA-Seq data. The first strand of cDNA was synthesized using ReverTra Ace qPCR RT Kit (Torobo Co., Osaka, Japan). qRT-PCR was carried out using an SYBR Green Real-time PCR Master Mix (Torobo Co., Osaka, Japan) according to the procedure: 94 °C for 5 min, then 35 cycles at 94 °C for 2 min and 55 °C for 30 s, and 72 °C for 30 s. Arz_12286_T1 gene encoding actin was used as an internal control for data acquisition and normalization. Primers for qRT-PCR of the marker genes are listed in Additional file 7: Table S1. The relative expression level of the marker genes was analyzed according to the method [67].

### HPLC and GC/MS methods

Glucose, xylose, and acetic acid were analyzed using HPLC (LC-20AD, refractive index detector RID-10A, Shimadzu, Kyoto, Japan) with Bio-Rad Aminex HPX-87H column at 0.6 mL/min of 5-mM sulfuric acid solution and the column temperature of 65 °C [14].

Furan and phenolic compounds were analyzed using reverse-phase HPLC (LC-20AT, SPD-20A UV detector, Shimadzu, Kyoto, Japan) equipped with YMC-Pack ODS-A column (YMC, Tokyo, Japan) [31]. All samples were filtered through the 0.22-μm membrane before HPLC analysis.

It identified the degradation intermediates of aldehyde inhibitors by *A. resinae* ZN1 on Agilent 6890 GC–MS (Agilent Technologies, Santa Clara, CA) with HP-5 MS column (30 m × 0.25 mm × 0.25 μm) from 80 °C (held for 4 min) to 280 °C at the rate of 8 °C/min. One microliter sample was detected under splitless condition [68, 69].

### Nucleotide sequence accession number

This Whole Genome Shotgun project reported in this paper has been deposited at DDBJ/EMBL/GenBank under the accession number JZSE00000000. The version described in this paper is the first version JZSE01000000. Raw reads of the WGS (Whole Genome Sequencing) sequencing have been deposited into the NCBI Sequence Read Archive (SRX908854 for WGS).

## Additional files

**Additional file 1: Figure S1.** Amplification of the 15 marker gene pairs in *A. resinae* ZN1.

**Additional file 2: Figure S2.** Genetic stability of *A. resinae* ZN1.

**Additional file 3: Figure S3.** Volcano plot of the differentially expressed genes (DEGs) during inhibitor degradation in *A. resinae* ZN1.

**Additional file 4: Dataset S1.** Predicted genes and their expression on the central carbon metabolism.

**Additional file 5: Figure S4.** Comparison of the expression of orthologous gene pairs in central metabolism.

**Additional file 6: Figure S5.** Gene ontology enrichment analysis of differentially expressed genes.

**Additional file 7: Table S1.** The primers used in this study.

## Data Availability

The data sets used and/or analyzed during the current study are available from the corresponding author on reasonable request.
