# Supplementary material for: Heterozygous diploid structure of Amorphotheca resinae ZN1 contributes efficient biodetoxification on solid pretreated corn stover
Source: Biotechnol Biofuels. 2019 May 21;12:126. doi: 10.1186/s13068-019-1466-z (PMC6528196; doi:10.1186/s13068-019-1466-z)
Supplement: Supplementary file 1 — Additional file 1: Figure S1. Amplification of the 15 marker gene pairs in A. resinae ZN1. [file 13068_2019_1466_MOESM1_ESM.docx]

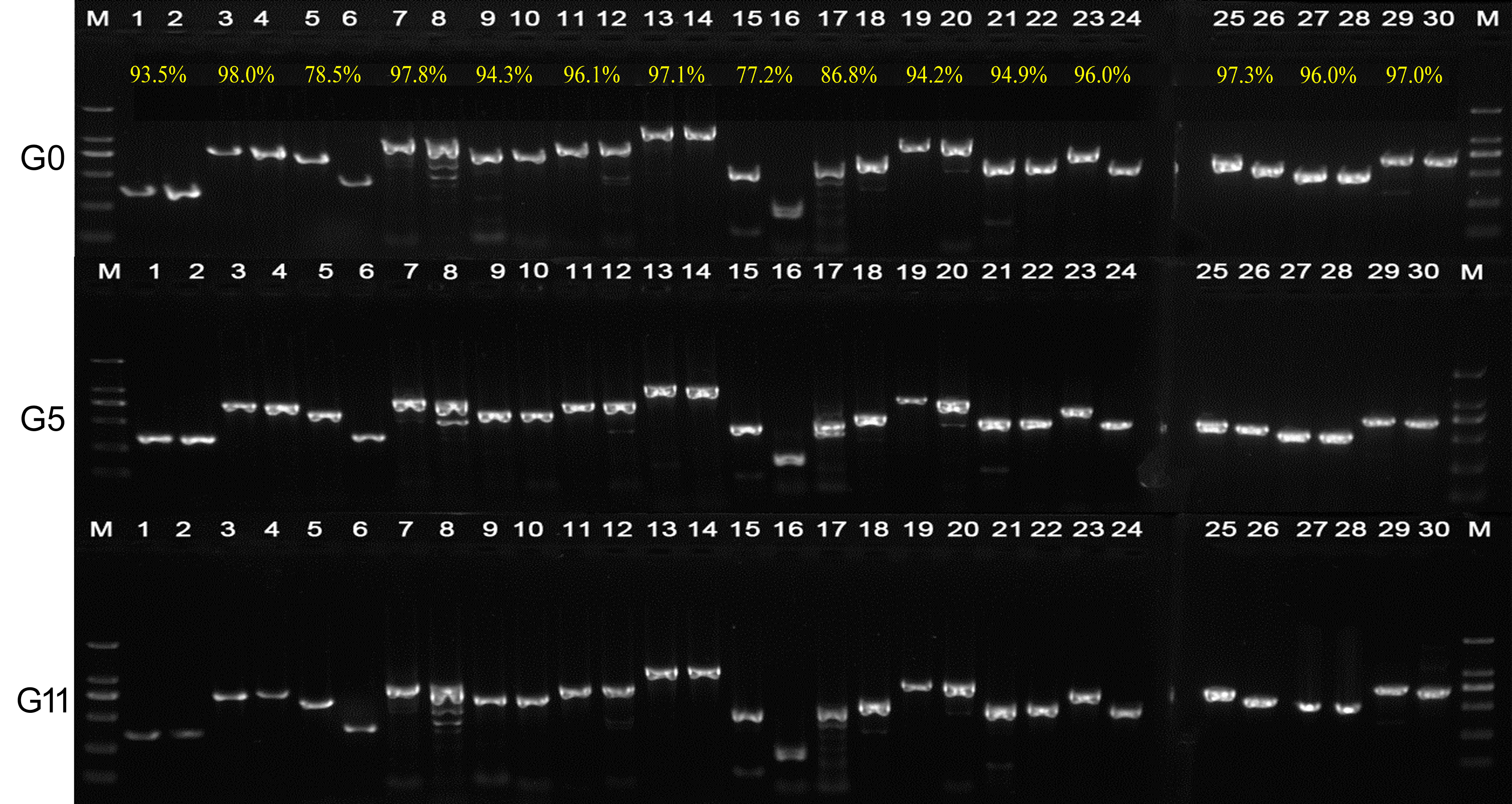


**Figure S1 Amplification of the fifteen marker gene pairs in *A. resinae* ZN1.** The genomic DNA templates were from the original (G0), the fifth (G5), and the eleventh transfers (G11) of *A. resinae* ZN1, respectively. The corresponding primers were listed in Additional file 6: Table S2. The amplified DNA fragments of each gene pairs were sequenced, and the homology similarity of overlapping regions was colored yellow. Lane 1, 1-ARZ_14322_T1; Lane 2, 1-ARZ_14430_T1; Lane 3, 2-ARZ_2135_T1; Lane 4, 2-ARZ_6929_T1; Lane 5, 3-ARZ_9744_T1; Lane 6, 3-ARZ_13904_T1; Lane 7, 4-ARZ_11534_T1; Lane 8, 4-ARZ_11870_T1; Lane 9, 5-ARZ_138_T1; Lane 10, 5-ARZ_6758_T1; Lane 11, 6-ARZ_14875_T1; Lane 12, 6-ARZ_17156_T1; Lane 13, 7-ARZ_7445_T1; Lane 14, 7-ARZ_11497_T1; Lane 15, 8-ARZ_581_T1; Lane 16, 8-ARZ_15233_T1; Lane 17, 9-ARZ_2411_T1; Lane 18, 9-ARZ_10881_T1; Lane 19, 10-ARZ_2545_T1; Lane 20, 10-ARZ_10290_T1; Lane 21, 11-ARZ_9991_T1; Lane 22, 11-ARZ_15400_T1; Lane 23, 12-ARZ_7271_T1; Lane 24, 12-ARZ_10682_T1; Lane 25, 13-ARZ_3780_T1; Lane 26, 13-ARZ_1809_T1; Lane 27, 14-ARZ_13700_T1; Lane 28, 14-ARZ_15925_T1; Lane 29, 15-ARZ_8414_T1; Lane 30, 15-ARZ_12452_T1; Lane M, DL 2000 DNA marker.
