# Supplementary material for: Heterozygous diploid structure of Amorphotheca resinae ZN1 contributes efficient biodetoxification on solid pretreated corn stover
Source: Biotechnol Biofuels. 2019 May 21;12:126. doi: 10.1186/s13068-019-1466-z (PMC6528196; doi:10.1186/s13068-019-1466-z)
Supplement: Supplementary file 2 — Additional file 2: Figure S2. Genetic stability of A. resinae ZN1. [file 13068_2019_1466_MOESM2_ESM.docx]

**Figure S2 Genetic stability of *A. resinae* ZN1.** G0 and G11 separately indicated the original and the 11^th^ transfer in the synthetic medium amended with single mode inhibitor.
