# Supplementary material for: Heterozygous diploid structure of Amorphotheca resinae ZN1 contributes efficient biodetoxification on solid pretreated corn stover
Source: Biotechnol Biofuels. 2019 May 21;12:126. doi: 10.1186/s13068-019-1466-z (PMC6528196; doi:10.1186/s13068-019-1466-z)
Supplement: Supplementary file 3 — Additional file 3: Figure S3. Volcano plot of the differentially expressed genes (DEGs) during inhibitor degradation in A. resinae ZN1. [file 13068_2019_1466_MOESM3_ESM.docx]

**Figure S3** **Volcano plot of the differential expressed genes (DEGs) during inhibitor degradation in *A. resinae* ZN1.** Red, green, and black dots separately indicate the differentially up-regulated, the differentially down-regulated, and the undifferentially regular expressed genes.
