# Supplementary material for: Heterozygous diploid structure of Amorphotheca resinae ZN1 contributes efficient biodetoxification on solid pretreated corn stover
Source: Biotechnol Biofuels. 2019 May 21;12:126. doi: 10.1186/s13068-019-1466-z (PMC6528196; doi:10.1186/s13068-019-1466-z)
Supplement: Supplementary file 4 — Additional file 4: Dataset S1. Predicted genes and their expression on the central carbon metabolism. [file 13068_2019_1466_MOESM4_ESM.pdf]

Dataset S1 Predicted genes and their expression on the central carbon metabolism.

FC, fold change; NA, not available; Inf, infinite; HBA, 4-Hydroxybenzaldehyde.

Gene symbol	Gene ID	Furfural		HMF		HBA		Vanillin		Syringaldehyde		Acetic acid		Annotation description
		FC	FDR	FC	FDR	FC	FDR	FC	FDR	FC	FDR	FC	FDR	
**Sugar transporter**														
	ARZ_5022_T1	1.1	8.38E-01	0.8	7.37E-01	1.1	8.97E-01	0.6	1.18E-01	0.5	1.06E-02	1.2	4.85E-01	sugar transporter STL1, 2.A.1.1.38
	ARZ_1365_T1	0.8	3.61E-01	1.1	8.60E-01	1.0	8.83E-01	0.8	5.22E-02	0.8	7.31E-02	1.1	4.63E-01	sugar transporter STL1, 2.A.1.1.38
	ARZ_17650_T1	1.1	9.46E-01	1.5	3.63E-01	1.8	3.28E-01	1.8	1.78E-01	1.4	5.69E-01	1.3	6.95E-03	sugar transporter STL1, 2.A.1.1.38
	ARZ_17375_T1	1.1	7.44E-01	1.3	5.31E-01	1.4	3.12E-01	0.7	3.10E-01	0.8	4.93E-01	0.8	1.47E-01	sugar transporter STL1, 2.A.1.1.38
	ARZ_15611_T1	1.5	1.54E-01	1.0	9.54E-01	1.1	8.93E-01	0.7	7.04E-01	0.4	7.83E-02	0.4	1.45E-06	sugar transporter STL1, 2.A.1.1.38
	ARZ_16613_T1	NA	NA	NA	NA	0.2	1.38E-01	0.4	2.80E-01	0.1	3.75E-02	NA	NA	sugar transporter STL1, 2.A.1.1.38
	ARZ_1364_T1	1.2	5.16E-01	1.3	3.47E-01	1.0	9.59E-01	0.7	1.10E-01	0.9	6.37E-01	0.6	3.49E-02	sugar transporter STL1, 2.A.1.1.38
	ARZ_9499_T1	4.1	5.86E-01	1.5	1.00E+00	2.1	7.83E-01	1.5	8.01E-01	0.7	9.43E-01	0.8	8.78E-01	sugar transporter STL1, 2.A.1.1.38
	ARZ_12262_T1	NA	NA	NA	NA	1.6	9.37E-01	0.9	9.11E-01	2.0	7.89E-01	2.5	3.11E-01	sugar transporter STL1, 2.A.1.1.38
	ARZ_11206_T1	0.9	5.77E-01	1.0	1.00E+00	0.9	9.21E-01	1.4	2.45E-01	1.2	6.54E-01	1.1	8.26E-01	sugar transporter STL1, 2.A.1.1.38
	ARZ_6588_T1	2.5	3.43E-14	1.6	8.65E-04	1.0	9.25E-01	1.6	2.38E-02	1.1	7.39E-01	0.7	1.05E-01	sugar transporter STL1, 2.A.1.1.38
	ARZ_13497_T1	0.7	6.23E-03	0.9	6.59E-01	1.1	4.84E-01	1.5	6.14E-04	1.1	3.02E-01	0.6	1.74E-01	sugar transporter STL1, 2.A.1.1.38
	ARZ_4770_T1	1.4	2.86E-02	1.2	4.09E-01	0.9	9.56E-01	1.2	6.07E-01	1.0	9.22E-01	1.3	7.65E-02	sugar transporter STL1, 2.A.1.1.38
	ARZ_12277_T1	NA	NA	NA	NA	0.0	9.25E-01	0.0	8.38E-01	0.0	8.74E-01	Inf	9.92E-01	sugar transporter STL1, 2.A.1.1.38
	ARZ_4519_T1	0.0	1.00E+00	0.0	1.00E+00	0.6	1.00E+00	1.2	1.00E+00	1.1	1.00E+00	Inf	9.92E-01	sugar transporter STL1, 2.A.1.1.38
	ARZ_3064_T1	NA	NA	NA	NA	Inf	7.65E-01	NA	NA	Inf	9.87E-01	0.0	1.00E+00	sugar transporter STL1, 2.A.1.1.38
	ARZ_11336_T1	NA	NA	Inf	8.92E-01	2.1	9.54E-01	4.0	7.08E-01	4.0	5.89E-01	0.6	5.51E-01	sugar transporter STL1, 2.A.1.1.38
	ARZ_9110_T1	1.0	1.00E+00	1.7	5.71E-01	0.7	4.75E-01	1.0	8.67E-01	1.0	9.70E-01	0.7	8.86E-01	sugar transporter STL1, 2.A.1.1.38
	ARZ_5201_T1	1.2	9.64E-01	0.4	4.15E-01	0.8	9.57E-01	1.2	9.27E-01	0.7	7.44E-01	0.6	5.19E-01	sugar transporter STL1, 2.A.1.1.38
	ARZ_17110_T1	1.4	9.16E-01	1.2	1.00E+00	2.2	2.84E-01	0.6	8.27E-01	2.6	7.30E-02	0.3	8.40E-02	sugar transporter STL1, 2.A.1.1.38
	ARZ_17814_T1	1.9	7.37E-01	3.6	1.77E-01	1.3	9.98E-01	0.4	6.93E-01	0.8	9.40E-01	0.3	7.76E-01	sugar transporter STL1, 2.A.1.1.38
	ARZ_18129_T1	1.1	1.00E+00	0.9	9.20E-01	0.8	7.91E-01	1.7	1.84E-01	0.7	5.36E-01	0.3	6.09E-02	putative uncharacterized protein STL1, 2.A.1.1.73
	ARZ_16564_T1	1.3	1.00E+00	0.0	1.00E+00	1.0	1.00E+00	0.0	3.06E-01	0.7	9.56E-01	1.9	7.47E-01	putative uncharacterized protein STL1, 2.A.1.1.73
	ARZ_13831_T1	1.2	1.00E+00	0.5	8.99E-01	0.0	1.00E+00	0.0	9.97E-01	0.0	1.00E+00	0.8	9.95E-01	putative uncharacterized protein STL1, 2.A.1.1.73
	ARZ_3393_T1	0.4	3.76E-06	1.1	8.28E-01	0.9	8.67E-01	0.5	6.75E-06	0.6	3.62E-03	0.4	4.33E-05	putative uncharacterized protein STL1, 2.A.1.1.73
	ARZ_1004_T1	0.4	1.83E-13	1.0	9.69E-01	1.0	8.11E-01	0.5	3.42E-21	0.7	4.72E-05	0.5	5.13E-06	putative uncharacterized protein STL1, 2.A.1.1.73
	ARZ_9061_T1	1.1	1.00E+00	0.0	1.00E+00	1.1	9.46E-01	0.4	1.80E-01	0.7	7.78E-01	0.0	1.00E+00	putative uncharacterized protein STL1, 2.A.1.1.73
	ARZ_15138_T1	0.6	7.02E-01	1.1	1.00E+00	0.8	8.63E-01	0.9	7.52E-01	0.8	7.11E-01	0.6	4.75E-01	putative uncharacterized protein STL1, 2.A.1.1.73
	ARZ_16871_T1	2.3	6.02E-25	1.7	1.34E-09	1.0	9.69E-01	1.0	9.76E-01	0.8	2.51E-01	0.3	1.48E-08	putative uncharacterized protein STL1, 2.A.1.1.73
	ARZ_7710_T1	2.4	2.58E-09	1.7	1.97E-03	1.3	3.35E-01	1.0	9.96E-01	0.7	1.41E-01	0.5	6.27E-12	putative uncharacterized protein STL1, 2.A.1.1.73
	ARZ_9498_T1	0.6	7.41E-01	1.1	1.00E+00	0.6	6.59E-01	1.1	9.42E-01	2.0	2.49E-01	0.7	6.09E-01	putative uncharacterized protein STL1, 2.A.1.1.73
	ARZ_18343_T1	0.9	6.81E-01	0.9	8.05E-01	1.4	1.74E-01	1.5	4.64E-02	1.4	1.44E-01	2.8	1.68E-13	putative uncharacterized protein STL1, 2.A.1.1.73
	ARZ_5564_T1	1.0	9.40E-01	0.9	1.50E-01	1.0	9.60E-01	1.0	9.50E-01	1.1	3.80E-01	0.9	3.10E-01	high-affinity glucose transporter, 2.A.1.1.39
	ARZ_7243_T1	1.0	9.30E-01	1.0	9.00E-01	1.2	9.40E-02	1.0	9.00E-01	1.2	6.90E-02	0.8	3.00E-03	high-affinity glucose transporter, 2.A.1.1.39

	ARZ_10823_T1	0.4	8.50E-03	1.2	8.40E-01	0.9	6.60E-01	1.2	4.80E-01	1.6	1.80E-02	0.2	3.50E-40	high-affinity glucose transporter, 2.A.1.1.39
	ARZ_2468_T1	0.5	1.30E-01	0.8	8.20E-01	1.0	9.60E-01	1.1	9.80E-01	1.5	1.70E-01	0.3	5.90E-03	high-affinity glucose transporter, 2.A.1.1.39
	ARZ_12900_T1	1.5	2.30E-01	1.0	1.00E+00	1.6	1.60E-02	0.8	3.60E-01	1.1	8.30E-01	1.6	4.40E-02	high-affinity glucose transporter, 2.A.1.1.39
	ARZ_12901_T1	1.2	8.60E-01	0.7	6.80E-01	1.6	6.10E-01	1.8	3.10E-01	1.3	7.60E-01	1.0	9.60E-01	high-affinity glucose transporter, 2.A.1.1.39
	ARZ_14039_T1	1.1	7.60E-01	1.1	8.30E-01	1.4	5.10E-01	0.6	1.10E-01	0.9	8.40E-01	2.0	1.60E-02	high-affinity glucose transporter, 2.A.1.1.39
	ARZ_18019_T1	0.0	7.10E-01	0.3	9.50E-01	0.0	7.60E-01	0.1	8.30E-01	2.4	8.50E-01	0.0	1.00E+00	high-affinity glucose transporter, 2.A.1.1.39
	ARZ_14863_T1	0.4	8.58E-01	0.0	5.22E-01	0.6	9.88E-01	0.3	5.10E-01	1.2	1.00E+00	0.2	6.06E-01	sugar transporter STL1, 2.A.1.1.38
	ARZ_14722_T1	0.6	8.60E-09	0.9	5.20E-01	0.8	1.00E-01	0.7	1.60E-03	1.3	5.20E-02	0.7	3.40E-02	monosaccharide transporter, 2.A.1.1.57
	ARZ_15507_T1	0.7	3.60E-03	1.2	9.90E-02	1.2	8.20E-01	2.0	2.20E-01	3.1	4.20E-02	0.5	1.80E-04	monosaccharide transporter, 2.A.1.1.57
	ARZ_18745_T1	NA	NA	NA	NA	NA	NA	NA	NA	Inf	8.30E-01	Inf	9.90E-01	monosaccharide transporter, 2.A.1.1.57
	ARZ_12681_T1	5.1	4.70E-02	2.3	5.20E-01	0.9	9.60E-01	1.5	8.20E-01	0.7	7.60E-01	0.4	2.60E-01	monosaccharide transporter, 2.A.1.1.58
	ARZ_12778_T1	2.4	1.40E-03	1.1	9.10E-01	1.4	5.20E-01	1.4	4.70E-01	2.1	7.70E-02	1.2	4.30E-01	monosaccharide transporter, 2.A.1.1.58
	ARZ_1852_T1	1.0	8.70E-01	1.3	3.90E-02	1.0	9.50E-01	0.8	4.90E-01	0.8	2.70E-01	0.7	1.60E-13	glucose transporter/sensor, 2.A.1.1.68
	ARZ_12158_T1	1.1	8.40E-01	1.2	3.00E-01	0.7	3.50E-01	0.6	5.70E-02	0.6	2.60E-02	0.8	3.00E-03	glucose transporter/sensor, 2.A.1.1.68
	ARZ_12566_T1	0.4	2.70E-08	0.7	7.90E-02	0.8	1.80E-02	0.7	7.10E-05	0.9	2.80E-01	0.8	1.80E-01	sugar/H+ symporter, 2.A.1.1.69
	ARZ_4608_T1	0.4	3.60E-03	0.7	3.60E-01	0.9	5.10E-01	0.7	1.80E-03	0.9	3.20E-01	1.1	7.20E-01	sugar/H+ symporter, 2.A.1.1.69
	ARZ_16926_T1	0.7	6.20E-04	1.0	8.80E-01	0.9	6.30E-01	0.4	1.00E-07	0.8	4.20E-01	0.6	5.30E-22	uncharacterized transporter, 2.A.1.2.46
	ARZ_18085_T1	0.7	5.80E-06	1.0	9.10E-01	0.8	1.70E-01	0.3	2.20E-21	0.5	1.60E-08	0.7	1.30E-03	uncharacterized transporter, 2.A.1.2.46
	ARZ_18689_T1	NA	NA	NA	NA	NA	NA	NA	NA	NA	NA	NA	NA	L-arabinose transport system permease protein araH, 3.A.1.2.2
	ARZ_18813_T1	NA	NA	NA	NA	NA	NA	NA	NA	NA	NA	NA	NA	L-arabinose-binding periplasmic protein precursor (ABP), 2.A.1.2.2
	ARZ_18803_T1	NA	NA	NA	NA	NA	NA	NA	NA	NA	NA	NA	NA	glucose/galactose transporter, 2.A.1.7.10
	ARZ_15900_T1	0.1	1.72E-04	0.4	1.77E-01	1.0	9.80E-01	0.8	4.13E-01	0.8	6.51E-01	0.8	9.92E-01	glucose/galactose transporter, 2.A.1.7.11
	ARZ_8567_T1	1.5	7.87E-01	0.6	8.05E-01	1.0	1.00E+00	0.8	6.85E-01	1.3	4.10E-01	0.3	4.46E-01	glucose/galactose transporter, 2.A.1.7.11
	ARZ_9169_T1	0.6	7.98E-06	0.9	5.40E-01	0.9	7.87E-01	0.9	8.34E-01	1.0	8.84E-01	0.3	1.90E-229	glucose/galactose transporter, 2.A.1.7.11
	ARZ_5265_T1	0.8	1.21E-01	1.1	8.29E-01	1.6	2.63E-03	1.1	3.53E-01	1.0	9.96E-01	0.4	5.31E-26	glucose/galactose transporter, 2.A.1.7.11
**Glycolysis and Gluconeogenesis**														
HK	ARZ_11412_T1	0.9	5.30E-01	0.9	8.50E-01	1.2	4.00E-04	1.0	6.70E-01	1.0	8.10E-01	0.7	4.10E-08	hexokinase
HK	ARZ_7354_T1	0.9	7.70E-01	0.8	2.10E-01	0.9	5.10E-01	1.3	1.40E-03	1.2	1.80E-02	0.7	8.20E-08	hexokinase
HK	ARZ_9863_T1	1.0	9.90E-01	1.1	3.60E-01	0.9	7.80E-01	0.7	1.20E-01	0.9	7.60E-01	0.5	2.50E-22	glucokinase
HK	ARZ_7815_T1	1.0	1.00E+00	1.1	6.40E-01	0.9	4.60E-01	1.0	6.60E-01	1.3	1.40E-03	0.6	1.70E-18	glucokinase
HK	ARZ_773_T1	0.4	1.40E-05	0.8	5.10E-01	0.9	5.10E-01	0.4	1.40E-12	0.6	1.40E-03	0.7	1.40E-03	hexokinase-1
GPI	ARZ_11045_T1	1.5	1.30E-07	1.4	6.40E-05	0.7	6.20E-02	1.1	5.50E-01	1.3	4.50E-02	0.5	9.20E-27	glucose-6-phosphate isomerase
GPI	ARZ_4835_T1	1.7	2.80E-19	1.6	5.50E-14	0.8	3.80E-02	1.2	8.20E-02	1.4	3.10E-06	0.6	3.40E-25	glucose-6-phosphate isomerase
PFK	ARZ_5995_T1	1.4	1.50E-05	1.1	2.50E-01	1.2	1.30E-01	1.2	5.30E-02	1.2	1.10E-01	1.0	9.60E-01	6-phosphofructokinase
PFK	ARZ_2927_T1	1.0	7.30E-01	1.0	7.80E-01	1.2	3.00E-01	1.2	1.30E-01	1.2	1.30E-01	0.9	4.90E-01	6-phosphofructokinase
FBA	ARZ_3943_T1	1.3	5.20E-02	1.1	8.00E-01	1.1	4.10E-01	1.1	2.30E-01	1.2	6.50E-03	0.9	4.00E-01	fructose-bisphosphate aldolase, class II
FBA	ARZ_8196_T1	1.4	1.10E-05	1.1	3.50E-01	0.9	3.70E-01	1.1	7.80E-01	1.2	4.70E-02	0.9	3.90E-02	fructose-bisphosphate aldolase, class II
TPI	ARZ_4465_T1	1.0	9.70E-01	0.9	7.70E-01	0.9	5.40E-01	1.0	9.50E-01	0.9	7.10E-01	1.0	8.30E-01	triosephosphate isomerase
TPI	ARZ_13243_T1	1.0	9.30E-01	1.0	1.00E+00	0.7	9.90E-03	0.9	1.50E-01	0.9	2.20E-01	1.5	5.50E-04	triosephosphate isomerase
TPI	ARZ_3119_T1	0.9	1.00E+00	0.9	1.00E+00	0.9	9.60E-01	1.3	5.70E-01	1.4	2.00E-01	0.8	8.00E-01	triosephosphate isomerase, putative

	TPI	ARZ_11286_T1	2.4	1.40E-01	1.3	9.20E-01	1.0	9.90E-01	0.7	2.00E-01	1.8	6.80E-02	1.3	6.60E-01	triosephosphate isomerase, putative
	GAPDH	ARZ_11978_T1	1.1	6.30E-08	1.3	2.90E-42	1.6	3.10E-04	1.2	4.10E-01	1.2	2.40E-01	1.1	7.50E-02	glyceraldehyde 3-phosphate dehydrogenase
	GAPDH	ARZ_7630_T1	1.0	1.00E+00	1.3	3.40E-02	1.5	1.70E-47	0.8	1.80E-09	0.9	2.40E-06	1.0	9.70E-01	glyceraldehyde 3-phosphate dehydrogenase
	PGK	ARZ_3920_T1	1.3	8.30E-03	1.2	8.10E-02	1.0	8.90E-01	1.1	6.90E-01	1.1	4.60E-01	1.0	9.00E-01	phosphoglycerate kinase
	PGK	ARZ_8173_T1	1.3	5.50E-05	1.2	1.30E-02	1.2	6.10E-01	1.5	1.00E-01	1.2	5.30E-01	0.9	4.50E-01	phosphoglycerate kinase
	PGAM	ARZ_5611_T1	1.0	5.00E-01	1.0	6.20E-01	1.1	7.00E-01	0.8	3.50E-01	0.9	6.00E-01	0.9	2.70E-01	phosphoglycerate mutase
	PGAM	ARZ_7197_T1	0.8	1.90E-04	0.9	2.20E-02	0.7	1.30E-02	1.0	7.00E-01	1.0	9.50E-01	1.2	6.90E-10	phosphoglycerate mutase
	PGAM	ARZ_9886_T1	1.6	1.20E-05	0.9	7.30E-01	0.8	2.60E-01	0.6	1.20E-03	0.8	2.10E-01	0.9	2.50E-01	phosphoglycerate mutase
	PGAM	ARZ_11645_T1	1.7	8.40E-10	1.1	6.00E-01	0.8	3.20E-01	0.9	4.00E-01	1.5	9.80E-03	0.8	4.30E-01	phosphoglycerate mutase
	PGAM	ARZ_5109_T1	1.5	1.90E-01	1.4	3.40E-01	1.2	8.10E-01	1.8	1.50E-01	2.1	5.90E-02	1.5	4.50E-07	phosphoglycerate mutase
	PGAM	ARZ_1445_T1	1.3	2.90E-02	1.0	9.50E-01	1.2	7.70E-01	1.7	8.10E-02	2.5	2.20E-03	1.7	2.00E-04	phosphoglycerate mutase
	ENO	ARZ_7843_T1	0.9	1.80E-01	1.1	4.70E-01	1.5	4.40E-02	1.2	3.60E-01	1.3	1.20E-01	0.9	6.30E-03	enolase
	ENO	ARZ_6306_T1	0.9	4.90E-01	1.1	8.70E-01	1.3	2.10E-01	1.3	2.40E-01	1.4	1.10E-01	0.9	1.10E-01	enolase
	PYK	ARZ_581_T1	0.7	3.70E-07	1.1	1.20E-01	0.9	6.40E-01	0.6	1.80E-02	1.0	9.60E-01	0.6	1.00E-12	pyruvate kinase
	PYK	ARZ_15233_T1	0.8	3.30E-03	1.3	6.10E-04	0.9	6.60E-01	0.6	1.50E-04	0.9	6.50E-01	0.7	1.10E-06	pyruvate kinase
	FBP	ARZ_4407_T1	2.4	1.20E-19	1.1	5.20E-01	1.6	8.60E-02	1.0	1.00E+00	1.0	9.90E-01	0.9	5.80E-01	fructose-1,6-bisphosphatase I
	FBP	ARZ_12961_T1	2.2	0.00E+00	0.9	1.60E-03	1.1	8.50E-01	0.7	1.50E-01	0.6	4.20E-02	1.2	3.00E-01	fructose-1,6-bisphosphatase I
	PEPCK	ARZ_3485_T1	1.7	1.10E-05	1.0	9.30E-01	1.5	4.90E-03	0.8	2.10E-01	0.8	1.70E-01	0.7	2.60E-01	phosphoenolpyruvate carboxykinase (ATP)
	PEPCK	ARZ_1094_T1	1.9	1.20E-80	1.3	8.00E-10	1.5	7.10E-02	0.6	3.50E-02	0.6	5.20E-02	0.6	1.30E-01	phosphoenolpyruvate carboxykinase (ATP)
	PEPCK	ARZ_3486_T1	1.5	8.80E-08	1.1	2.20E-01	1.8	9.90E-03	0.5	1.10E-03	0.5	1.60E-04	1.0	9.60E-01	phosphoenolpyruvate carboxykinase (ATP)
	PEPCK	ARZ_1095_T1	1.8	2.30E-08	1.2	2.90E-01	1.6	9.90E-02	0.5	4.00E-03	0.6	4.30E-02	0.6	9.20E-02	phosphoenolpyruvate carboxykinase (ATP)
	PC	ARZ_14322_T1	1.0	4.20E-01	1.3	1.10E-13	1.4	5.40E-15	1.0	3.60E-01	1.3	9.40E-10	0.8	1.70E-26	pyruvate carboxylase
	PC	ARZ_14430_T1	0.8	1.10E-02	1.2	4.10E-02	1.3	3.60E-05	0.8	8.90E-05	1.3	1.30E-04	0.8	1.30E-43	pyruvate carboxylase
**Pentose phosphate pathway**															
	GPDH	ARZ_9744_T1	1.4	7.20E-24	1.5	1.80E-27	1.1	3.10E-01	1.4	1.50E-05	1.7	2.00E-15	0.6	1.80E-15	glucose-6-phosphate 1-dehydrogenase
	GPDH	ARZ_13904_T1	2.0	4.40E-65	1.5	4.30E-21	1.1	3.90E-02	1.2	8.90E-07	1.4	3.80E-26	0.7	2.00E-03	glucose-6-phosphate 1-dehydrogenase
	PGL	ARZ_13072_T1	1.6	8.40E-04	1.2	5.40E-01	1.0	8.80E-01	0.8	4.10E-01	1.0	1.00E+00	1.3	4.20E-02	6-phosphogluconolactonase
	PGL	ARZ_15802_T1	1.5	5.50E-04	1.3	7.40E-02	0.8	5.40E-02	0.9	1.90E-01	1.0	9.90E-01	1.0	9.50E-01	6-phosphogluconolactonase
	PGD	ARZ_11446_T1	1.7	1.30E-11	1.4	4.50E-05	1.4	1.80E-03	0.8	5.20E-03	0.7	1.20E-04	1.0	6.80E-01	6-phosphogluconate dehydrogenase
	PGD	ARZ_7395_T1	1.5	8.60E-51	1.3	8.60E-16	1.2	1.80E-02	0.6	2.40E-20	0.6	4.20E-18	1.1	1.70E-02	6-phosphogluconate dehydrogenase
	PGD	ARZ_6146_T1	1.0	1.00E+00	0.9	7.80E-01	1.1	8.90E-01	0.6	4.50E-02	1.4	2.90E-01	0.3	3.20E-11	6-phosphogluconate dehydrogenase
	PGD	ARZ_10060_T1	1.0	9.60E-01	0.8	6.60E-01	1.2	8.30E-01	1.0	1.00E+00	1.6	3.10E-01	0.7	4.90E-03	6-phosphogluconate dehydrogenase
	RPE	ARZ_1979_T1	1.5	6.50E-10	1.2	2.90E-02	1.3	4.30E-01	1.0	8.90E-01	1.3	3.60E-01	1.1	7.80E-01	ribulose-phosphate 3-epimerase
	RPE	ARZ_4254_T1	1.4	4.60E-02	1.1	6.10E-01	1.1	6.90E-01	1.0	9.80E-01	1.1	6.20E-01	0.9	7.90E-01	ribulose-phosphate 3-epimerase
	RPI	ARZ_9929_T1	1.2	3.20E-02	1.1	3.40E-01	1.0	9.80E-01	1.0	1.00E+00	1.4	1.20E-01	1.2	5.80E-02	ribose 5-phosphate isomerase A
	RPI	ARZ_18006_T1	1.2	2.10E-01	1.1	6.50E-01	0.8	7.00E-01	1.6	1.70E-01	1.8	5.00E-02	1.1	7.50E-01	ribose 5-phosphate isomerase A
	RPI	ARZ_3120_T1	1.0	1.00E+00	1.3	8.30E-01	0.9	9.80E-01	0.8	9.90E-01	1.0	1.00E+00	1.5	4.60E-01	ribose 5-phosphate isomerase B
	RPI	ARZ_11285_T1	0.8	5.70E-01	0.6	1.20E-01	0.6	2.70E-01	0.4	3.80E-02	0.8	6.10E-01	1.0	1.00E+00	ribose 5-phosphate isomerase B
	TK	ARZ_6724_T1	1.4	1.30E-06	1.3	7.70E-04	1.3	2.30E-02	1.2	8.10E-02	1.3	2.80E-02	0.7	4.70E-07	transketolase
	TK	ARZ_99_T1	1.3	7.50E-02	1.2	2.10E-01	1.6	4.60E-15	1.2	1.90E-03	1.3	2.10E-05	0.6	1.40E-10	transketolase

TA	ARZ_6051_T1	1.7	1.50E-171	1.1	1.80E-03	1.2	3.50E-03	1.3	5.30E-06	1.5	5.70E-12	0.7	2.30E-06	transaldolase
TA	ARZ_2982_T1	1.9	1.40E-16	1.0	1.00E+00	1.2	8.90E-02	1.2	7.30E-02	1.2	7.40E-02	0.8	2.50E-02	transaldolase
**Glycerol metabolism**														
GPD	ARZ_9114_T1	0.4	1.10E-48	0.9	4.10E-01	0.6	8.50E-04	0.7	3.90E-02	0.8	3.60E-01	0.7	9.20E-05	glycerol-3-phosphate dehydrogenase (NAD+)
GPD	ARZ_5204_T1	0.6	7.60E-09	1.0	1.00E+00	0.9	4.00E-01	1.0	6.10E-01	1.0	9.30E-01	0.9	4.90E-01	glycerol-3-phosphate dehydrogenase (NAD+)
GPD	ARZ_17921_T1	0.9	2.00E-01	0.9	2.20E-01	0.9	6.50E-01	0.7	1.10E-03	0.9	1.60E-01	1.2	4.10E-15	glycerol-3-phosphate dehydrogenase, mitochondrial
GPD	ARZ_17787_T1	0.6	2.30E-07	0.8	1.70E-02	0.8	2.60E-02	1.2	2.20E-01	1.0	8.80E-01	1.3	5.50E-02	glycerol-3-phosphate dehydrogenase, mitochondrial
GPP	ARZ_3959_T1	0.9	6.10E-01	1.2	2.30E-01	1.1	3.70E-01	0.6	3.50E-06	0.7	1.80E-03	1.6	2.40E-02	glycerol 3-phosphatase 1
GPP	ARZ_8212_T1	1.0	9.80E-01	1.2	5.90E-01	1.4	5.60E-01	0.6	1.30E-01	0.6	1.10E-01	2.2	3.50E-21	glycerol 3-phosphatase 1
GLK	ARZ_4453_T1	0.7	1.70E-05	0.8	2.40E-02	0.8	5.80E-01	0.7	1.00E-01	0.8	4.10E-01	0.8	9.00E-18	glycerol kinase
GLK	ARZ_13256_T1	0.7	5.00E-06	0.8	4.60E-02	0.7	2.20E-03	0.6	3.50E-05	0.7	2.20E-04	0.8	1.60E-09	glycerol kinase
DHAK	ARZ_10857_T1	0.8	2.60E-02	1.0	8.80E-01	0.8	5.50E-05	0.7	3.40E-08	0.8	4.50E-04	0.5	4.30E-12	dihydroxyacetone kinase
DHAK	ARZ_2435_T1	0.9	2.10E-01	0.9	7.00E-01	0.7	4.40E-18	0.7	1.00E-31	0.7	2.70E-18	0.4	7.40E-15	dihydroxyacetone kinase
DHAK	ARZ_3043_T1	1.4	6.50E-01	1.0	1.00E+00	1.2	7.60E-01	1.0	9.00E-01	0.9	9.00E-01	1.4	3.90E-01	dihydroxyacetone kinase
DHAK	ARZ_11362_T1	0.4	7.30E-02	0.9	1.00E+00	1.2	1.00E-01	1.0	1.00E+00	0.7	1.30E-02	1.6	7.80E-02	dihydroxyacetone kinase
DHAK	ARZ_3044_T1	0.0	8.80E-01	3.3	9.00E-01	1.0	9.90E-01	1.1	7.50E-01	1.1	7.50E-01	0.2	4.50E-01	dihydroxyacetone kinase
DHAK	ARZ_11360_T1	0.0	1.00E+00	0.0	1.00E+00	1.2	2.70E-01	1.3	6.10E-02	1.3	7.50E-02	0.2	6.00E-01	dihydroxyacetone kinase
DHAK	ARZ_2922_T1	NA	NA	Inf	1.00E+00	1.6	6.40E-01	0.9	1.00E+00	0.4	2.90E-01	NA	NA	dihydroxyacetone kinase
GLD	ARZ_8147_T1	1.3	4.80E-01	1.1	1.00E+00	1.4	4.40E-01	1.4	4.10E-01	0.9	7.80E-01	1.5	7.30E-02	glycerol dehydrogenase
GLD	ARZ_2573_T1	1.1	3.80E-01	0.9	4.10E-01	1.9	4.70E-03	1.2	5.20E-01	1.0	9.80E-01	1.0	9.30E-01	glycerol dehydrogenase
**TCA cycle**														
PDH	ARZ_16974_T1	2.0	6.30E-34	1.4	7.20E-07	1.6	2.50E-06	1.2	8.00E-02	1.1	4.80E-01	1.0	7.30E-01	pyruvate dehydrogenase E1 component subunit alpha
PDH	ARZ_16047_T1	1.8	2.70E-07	1.1	4.90E-01	1.4	4.20E-07	1.2	3.00E-02	1.1	1.10E-01	1.4	3.00E-04	pyruvate dehydrogenase E1 component subunit alpha
PDH	ARZ_16517_T1	1.4	3.20E-04	1.0	1.00E+00	1.2	5.50E-07	1.1	5.60E-04	0.8	3.50E-05	1.3	1.70E-14	dihydrolipoamide dehydrogenase
PDH	ARZ_7525_T1	1.5	7.10E-12	0.9	3.20E-01	1.4	1.40E-02	1.0	8.30E-01	0.8	7.60E-02	1.7	6.80E-16	dihydrolipoamide dehydrogenase
PDH	ARZ_6001_T1	1.9	7.90E-298	1.6	6.40E-127	1.5	1.40E-01	1.3	2.70E-01	1.3	2.90E-01	1.0	9.80E-01	pyruvate dehydrogenase E1 component subunit beta
PDH	ARZ_2933_T1	2.0	6.80E-23	1.5	1.50E-08	1.3	6.30E-02	1.2	2.90E-01	1.0	9.50E-01	1.3	4.70E-02	pyruvate dehydrogenase E1 component subunit beta
PDH	ARZ_14710_T1	1.4	1.30E-37	1.2	4.70E-19	1.1	4.50E-01	1.0	9.50E-01	1.0	9.80E-01	1.1	1.50E-01	pyruvate dehydrogenase E2 component (dihydrolipoamide acetyltransferase)
PDH	ARZ_15521_T1	1.5	5.30E-08	1.2	3.50E-02	1.4	1.10E-01	1.0	9.80E-01	0.9	6.20E-01	1.3	1.00E-05	pyruvate dehydrogenase E2 component (dihydrolipoamide acetyltransferase)
PDH	ARZ_17747_T1	1.7	8.20E-05	1.1	6.40E-01	0.9	7.60E-01	1.2	2.10E-01	1.0	8.10E-01	0.8	2.50E-01	pyruvate dehydrogenase E1 component subunit alpha
CS	ARZ_17756_T1	1.3	1.60E-02	1.2	2.20E-01	1.3	4.20E-01	1.5	1.50E-01	1.5	1.60E-01	2.2	3.80E-04	citrate synthase, mitochondrial
CS	ARZ_7305_T1	1.5	3.20E-04	1.1	5.30E-01	1.3	2.60E-01	1.8	6.50E-03	1.4	1.00E-01	1.8	3.60E-05	citrate synthase, mitochondrial
CS	ARZ_11892_T1	1.4	5.00E-08	1.1	1.30E-01	0.9	4.10E-01	1.1	4.60E-01	1.1	3.60E-01	0.9	1.10E-01	2-methylcitrate synthase, mitochondrial
CS	ARZ_11559_T1	1.0	7.30E-01	1.0	7.60E-01	0.8	1.30E-01	1.1	5.50E-01	1.1	6.00E-01	0.6	3.40E-24	2-methylcitrate synthase, mitochondrial
ACO	ARZ_15054_T1	1.4	3.00E-06	1.2	7.90E-02	1.2	2.40E-01	1.0	6.30E-01	1.1	7.60E-01	1.7	2.30E-12	aconitate hydratase, mitochondrial
ACO	ARZ_8656_T1	1.3	5.70E-04	1.1	3.50E-01	1.3	4.10E-02	1.1	3.70E-01	1.1	4.90E-01	1.7	6.00E-18	aconitate hydratase, mitochondrial
ACO	ARZ_17841_T1	1.4	4.80E-20	1.1	5.40E-02	1.0	9.00E-01	1.0	9.30E-01	1.2	1.70E-01	1.0	8.50E-01	aconitate hydratase 2, putative
ACO	ARZ_16685_T1	1.5	6.80E-04	1.2	2.50E-01	1.2	6.30E-01	1.2	5.20E-01	1.4	1.00E-01	1.0	1.00E+00	aconitate hydratase 2, putative

ACO	ARZ_18645_T1	1.3	3.40E-02	1.1	8.10E-01	1.4	8.20E-02	1.0	7.00E-01	1.2	2.00E-01	1.0	8.60E-01	aconitate hydratase 2, putative
IDH	ARZ_5863_T1	2.3	1.10E-03	1.1	8.40E-01	1.2	3.30E-01	1.0	9.30E-01	0.8	4.50E-01	3.0	1.10E-10	isocitrate dehydrogenase (NADP+), mitochondrial
IDH	ARZ_445_T1	1.4	1.10E-01	1.0	1.00E+00	1.2	1.10E-01	0.6	3.70E-10	0.8	4.90E-04	3.3	4.90E-82	isocitrate dehydrogenase (NADP+), mitochondrial
IDH	ARZ_9076_T1	1.7	4.90E-12	1.2	1.00E-01	1.0	9.80E-01	2.0	3.80E-02	1.7	1.30E-01	1.4	3.00E-03	isocitrate dehydrogenase (NAD+) subunit 1, mitochondrial
IDH	ARZ_5166_T1	1.4	7.40E-03	0.9	4.80E-01	1.3	4.60E-01	1.6	4.30E-02	1.4	1.60E-01	1.8	2.10E-23	isocitrate dehydrogenase (NAD+) subunit 1, mitochondrial
IDH	ARZ_15969_T1	1.3	1.20E-07	0.9	3.10E-01	2.3	2.70E-09	1.2	1.60E-01	0.9	6.70E-01	1.9	4.50E-08	isocitrate dehydrogenase (NAD+) subunit 2, mitochondrial
IDH	ARZ_4883_T1	1.5	3.10E-05	1.1	3.40E-01	2.1	7.10E-12	1.2	2.20E-01	0.8	1.30E-01	1.7	1.10E-05	isocitrate dehydrogenase (NAD+) subunit 2, mitochondrial
OGDH	ARZ_13302_T1	1.5	8.40E-13	1.2	1.20E-02	1.2	7.80E-02	1.3	2.10E-02	1.0	9.00E-01	1.2	4.00E-03	2-oxoglutarate dehydrogenase E1 component
OGDH	ARZ_4130_T1	1.6	3.90E-10	1.3	3.10E-03	1.6	2.10E-07	1.7	4.20E-09	1.3	1.10E-03	1.0	6.00E-01	2-oxoglutarate dehydrogenase E1 component
OGDH	ARZ_16491_T1	1.8	1.50E-13	1.1	6.20E-01	1.4	2.40E-14	1.2	3.10E-04	0.9	2.50E-03	1.5	4.90E-07	2-oxoglutarate dehydrogenase E2 component
OGDH	ARZ_7499_T1	1.8	1.40E-11	1.0	8.60E-01	1.5	9.50E-10	1.6	8.20E-13	1.1	9.00E-02	1.2	1.20E-01	2-oxoglutarate dehydrogenase E2 component
OGDH	ARZ_16517_T1	1.4	3.20E-04	1.0	1.00E+00	1.2	5.50E-07	1.1	5.60E-04	0.8	3.50E-05	1.3	1.70E-14	dihydrolipoamide dehydrogenase
OGDH	ARZ_7525_T1	1.5	7.10E-12	0.9	3.20E-01	1.4	1.40E-02	1.0	8.30E-01	0.8	7.60E-02	1.7	6.80E-16	dihydrolipoamide dehydrogenase
SCS	ARZ_11668_T1	6.0	3.00E-18	1.5	1.60E-01	1.3	5.30E-02	1.1	5.70E-01	1.1	5.40E-01	1.9	2.00E-10	succinyl-CoA synthetase alpha subunit
SCS	ARZ_12480_T1	5.9	3.70E-35	1.6	9.00E-03	1.3	1.20E-04	1.0	4.70E-01	0.9	8.80E-02	1.4	1.30E-01	succinyl-CoA synthetase alpha subunit
SCS	ARZ_3530_T1	1.1	1.10E-04	0.8	2.60E-16	2.6	1.80E-19	1.4	1.30E-03	0.9	5.50E-01	1.3	7.60E-03	succinyl-CoA synthetase alpha subunit
SCS	ARZ_16173_T1	1.1	8.20E-01	0.7	8.50E-02	2.7	8.80E-36	1.7	4.60E-10	1.0	1.00E+00	1.4	2.60E-03	succinyl-CoA synthetase alpha subunit
SCS	ARZ_10191_T1	2.1	3.50E-01	1.8	6.00E-01	1.7	9.40E-02	2.2	4.00E-04	2.0	6.00E-03	0.9	8.90E-01	succinyl-CoA synthetase alpha subunit
SCS	ARZ_15744_T1	1.4	4.80E-01	1.1	9.60E-01	1.0	1.00E+00	0.9	6.10E-01	1.5	3.50E-01	3.3	2.90E-15	succinyl-CoA synthetase alpha subunit
SCS	ARZ_16239_T1	1.1	1.70E-01	0.9	1.00E-01	2.6	2.10E-13	2.3	5.40E-11	1.6	7.90E-04	1.1	4.80E-01	succinyl-CoA synthetase beta subunit
SCS	ARZ_15157_T1	1.1	2.00E-01	0.9	5.00E-01	2.0	7.00E-07	1.9	2.00E-06	1.4	2.90E-02	0.9	1.80E-03	succinyl-CoA synthetase beta subunit
SCS	ARZ_10192_T1	5.8	4.60E-01	0.0	1.00E+00	1.8	2.70E-01	1.5	3.50E-01	1.8	2.00E-01	0.0	4.60E-01	succinyl-CoA synthetase beta subunit
SCS	ARZ_15743_T1	2.8	2.20E-13	0.7	1.80E-01	1.1	9.30E-01	1.7	1.90E-01	1.4	5.10E-01	1.0	1.00E+00	succinyl-CoA synthetase beta subunit
SDH	ARZ_5502_T1	1.4	8.80E-03	0.7	1.70E-02	0.4	2.30E-21	0.6	1.30E-06	0.4	3.00E-24	3.1	7.40E-10	succinate dehydrogenase (ubiquinone) cytochrome b subunit
SDH	ARZ_11779_T1	1.3	1.60E-01	0.7	5.40E-02	0.4	1.60E-05	0.7	1.70E-01	0.5	4.60E-04	2.2	2.10E-07	succinate dehydrogenase (ubiquinone) cytochrome b subunit
SDH	ARZ_1652_T1	1.7	4.70E-07	1.0	1.00E+00	1.0	1.00E+00	1.0	7.90E-01	1.0	9.10E-01	1.6	2.30E-03	succinate dehydrogenase (ubiquinone) membrane anchor subunit
SDH	ARZ_3623_T1	1.8	1.20E-06	1.1	5.30E-01	1.2	1.80E-01	0.7	1.60E-02	0.6	1.80E-04	2.6	1.60E-11	succinate dehydrogenase (ubiquinone) membrane anchor subunit
SDH	ARZ_16923_T1	2.0	2.90E-06	1.1	6.80E-01	1.2	4.20E-01	0.9	4.20E-01	0.8	1.10E-01	1.9	9.10E-243	succinate dehydrogenase (ubiquinone) iron-sulfur protein
SDH	ARZ_18080_T1	2.1	2.60E-20	1.2	5.70E-02	1.1	4.60E-01	0.8	1.70E-02	0.6	7.10E-06	2.0	5.40E-10	succinate dehydrogenase (ubiquinone) iron-sulfur protein
SDH	ARZ_1957_T1	2.4	3.90E-34	1.0	1.00E+00	1.1	6.60E-02	1.1	1.70E-01	0.9	3.80E-02	1.5	3.60E-06	succinate dehydrogenase (ubiquinone) flavoprotein subunit
SDH	ARZ_4232_T1	2.4	1.50E-17	1.0	1.00E+00	1.4	1.10E-06	1.3	6.50E-06	1.2	1.20E-02	1.4	3.30E-06	succinate dehydrogenase (ubiquinone) flavoprotein subunit
SDH	ARZ_3292_T1	1.8	2.40E-06	2.1	4.70E-09	0.8	1.80E-03	1.5	1.90E-09	1.1	4.90E-01	0.9	6.40E-01	succinate dehydrogenase (ubiquinone) flavoprotein subunit
SDH	ARZ_16736_T1	1.0	9.70E-01	2.0	2.10E-04	1.0	9.30E-01	2.1	1.00E-12	1.7	7.00E-07	0.9	1.10E-01	succinate dehydrogenase (ubiquinone) flavoprotein subunit
SDH	ARZ_4230_T1	1.0	9.30E-01	1.0	9.10E-01	0.9	1.10E-01	1.3	3.00E-04	1.0	9.70E-01	0.6	3.60E-29	succinate dehydrogenase (ubiquinone) flavoprotein subunit
FUM	ARZ_11189_T1	1.4	2.50E-07	1.0	9.90E-01	1.7	1.50E-14	0.9	1.30E-01	0.7	4.80E-09	2.1	1.60E-33	fumarate hydratase, class II
FUM	ARZ_6607_T1	1.3	8.30E-108	0.8	6.60E-34	1.3	6.60E-17	1.3	3.50E-11	1.0	4.20E-01	1.4	5.70E-03	fumarate hydratase, class II
MDH	ARZ_17272_T1	1.5	1.90E-03	1.1	5.80E-01	1.1	8.80E-01	1.5	5.30E-02	1.4	1.50E-01	1.8	2.30E-09	malate dehydrogenase, mitochondrial
MDH	ARZ_16701_T1	1.5	1.50E-40	1.1	8.00E-04	1.1	8.30E-01	1.2	3.00E-01	1.1	6.70E-01	2.3	4.80E-07	malate dehydrogenase, mitochondrial
**Glyoxylate cycle**														
CS	ARZ_18246_T1	1.2	1.00E+00	1.0	1.00E+00	1.4	9.40E-01	1.1	1.00E+00	1.7	7.90E-01	3.3	6.30E-02	citrate synthase 2, peroxisomal

CS	ARZ_15753_T1	1.3	1.00E+00	0.0	1.00E+00	2.3	9.80E-01	2.6	8.00E-01	0.0	1.00E+00	0.0	2.60E-01	citrate synthase 2, peroxisomal
CS	ARZ_18245_T1	0.0	1.00E+00	0.0	1.00E+00	Inf	9.80E-01	NA	NA	Inf	9.70E-01	Inf	1.00E+00	citrate synthase 2, peroxisomal
CS	ARZ_15754_T1	0.0	4.70E-01	0.3	8.60E-01	1.9	9.30E-01	2.7	6.10E-01	3.0	5.70E-01	0.0	1.00E+00	citrate synthase 2, peroxisomal
ICL	ARZ_5880_T1	1.1	1.40E-04	1.2	1.10E-07	0.8	4.90E-01	0.8	3.30E-01	1.1	4.80E-01	1.2	1.60E-02	isocitrate lyase
ICL	ARZ_2750_T1	1.2	1.20E-02	1.2	3.60E-02	0.7	2.90E-01	0.7	2.50E-01	0.9	7.10E-01	1.4	2.10E-04	isocitrate lyase
ICL	ARZ_11893_T1	1.2	2.30E-03	1.2	2.50E-02	1.0	9.80E-01	1.1	3.90E-01	1.1	2.50E-01	0.6	3.90E-07	mitochondrial 2-methylisocitrate lyase
ICL	ARZ_11560_T1	1.1	4.60E-03	1.1	3.50E-02	1.1	2.70E-02	1.0	9.20E-01	1.0	8.30E-01	0.6	3.80E-04	mitochondrial 2-methylisocitrate lyase
MDH	ARZ_9544_T1	1.3	4.30E-03	1.1	3.80E-01	1.0	1.00E+00	0.9	4.90E-07	0.9	2.50E-05	1.2	4.10E-01	malate dehydrogenase, cytoplasmic
MDH	ARZ_1179_T1	1.6	2.00E-16	1.2	3.90E-02	1.1	1.70E-01	0.8	5.80E-02	0.9	1.70E-01	1.2	3.30E-01	malate dehydrogenase, cytoplasmic
MS	ARZ_16872_T1	1.5	4.30E-04	1.3	7.30E-02	1.1	6.30E-01	0.7	1.60E-03	0.6	1.60E-05	1.1	5.70E-01	malate synthase, glyoxysomal
MS	ARZ_7709_T1	1.4	4.40E-18	1.3	3.00E-10	1.0	9.70E-01	0.6	1.30E-04	0.6	5.50E-07	1.0	1.00E+00	malate synthase, glyoxysomal
**Xylose and Arabinose metabolism**														
XR	ARZ_17678_T1	2.1	4.42E-03	1.2	6.64E-01	0.8	5.54E-01	1.2	5.74E-01	0.9	8.34E-01	0.9	5.18E-01	NAD(P)H-dependent D-xylose reductase
XR	ARZ_17792_T1	1.7	1.94E-01	0.9	8.57E-01	0.8	6.91E-01	2.0	3.53E-02	1.6	1.27E-01	1.0	8.69E-01	NAD(P)H-dependent D-xylose reductase
XR	ARZ_1621_T1	0.7	2.02E-23	0.7	6.34E-20	1.1	6.64E-01	0.9	4.23E-01	0.9	5.36E-01	1.2	8.94E-02	NAD(P)H-dependent D-xylose reductase
XR	ARZ_3591_T1	0.7	2.34E-04	0.7	1.55E-04	1.0	9.91E-01	1.0	6.17E-01	1.1	1.12E-01	1.3	2.73E-02	NAD(P)H-dependent D-xylose reductase
XDH	ARZ_16075_T1	0.6	1.67E-01	0.8	7.97E-01	0.4	2.02E-05	0.5	2.29E-04	0.6	1.55E-02	1.4	4.64E-02	xylitol dehydrogenase
XDH	ARZ_9244_T1	1.0	1.00E+00	1.1	7.24E-01	0.8	7.46E-03	0.7	3.13E-05	0.5	3.93E-13	1.0	9.14E-01	xylitol dehydrogenase
LAD	ARZ_16054_T1	2.1	9.33E-21	0.7	2.64E-02	1.3	4.25E-02	1.4	3.28E-03	1.0	9.51E-01	0.9	7.50E-01	L-arabinitol 4-dehydrogenase
LAD	ARZ_9265_T1	2.4	1.04E-26	0.9	3.33E-01	1.3	4.77E-01	1.0	9.48E-01	1.3	3.97E-01	0.8	4.45E-01	L-arabinitol 4-dehydrogenase
LXR	ARZ_11219_T1	1.0	9.87E-01	1.3	2.88E-01	1.1	3.50E-01	0.7	1.77E-03	0.6	6.88E-08	1.1	2.62E-01	L-xylulose reductase
LXR	ARZ_6576_T1	1.4	6.26E-10	1.1	1.13E-01	1.5	2.13E-03	0.6	1.32E-03	0.8	1.66E-01	0.9	8.18E-01	L-xylulose reductase
LXR	ARZ_578_T1	0.6	7.24E-01	0.4	4.48E-01	1.3	5.39E-01	1.4	2.57E-01	1.6	1.08E-01	0.8	5.79E-01	L-xylulose reductase
LXR	ARZ_15237_T1	1.5	1.34E-01	0.9	7.94E-01	1.4	3.73E-02	1.2	1.16E-01	1.2	1.95E-01	0.6	2.55E-01	L-xylulose reductase
XK	ARZ_15682_T1	1.1	8.81E-01	1.4	5.92E-02	0.7	3.83E-04	1.1	1.79E-01	1.1	1.29E-01	1.4	1.21E-03	xylulokinase
XK	ARZ_13958_T1	1.3	1.52E-02	1.4	1.78E-02	1.2	8.40E-01	2.5	4.56E-02	2.4	6.61E-02	1.0	1.00E+00	xylulokinase
**Mannose metabolism**														
MPI	ARZ_12290_T1	1.1	7.38E-02	1.2	2.61E-04	0.8	2.72E-01	1.1	6.12E-01	1.1	4.50E-01	1.0	7.14E-01	mannose-6-phosphate isomerase
MPI	ARZ_4533_T1	0.9	1.00E-01	1.0	1.00E+00	0.7	1.63E-01	1.2	6.39E-01	1.0	8.99E-01	1.0	9.87E-01	mannose-6-phosphate isomerase
MPI	ARZ_4722_T1	1.4	1.59E-01	1.0	1.00E+00	1.2	5.86E-01	1.4	2.81E-01	1.7	4.77E-02	0.2	1.70E-02	mannose-6-phosphate isomerase
MPI	ARZ_17723_T1	0.9	3.95E-01	0.8	2.89E-01	1.1	9.23E-01	1.6	6.61E-01	1.4	6.90E-01	0.2	4.48E-03	mannose-6-phosphate isomerase
**Galactose metabolism**														
GALK	ARZ_6798_T1	1.3	1.61E-03	1.3	1.39E-03	0.9	2.31E-01	0.8	4.81E-04	0.9	2.74E-01	1.1	6.07E-01	galactokinase
GALK	ARZ_176_T1	1.0	1.00E+00	1.4	4.92E-01	1.1	6.22E-01	0.6	3.97E-02	0.6	8.82E-03	1.7	6.01E-09	galactokinase
GALT	ARZ_11052_T1	1.7	2.11E-09	1.1	3.53E-01	1.1	4.29E-01	1.2	8.55E-04	1.3	2.88E-05	1.3	1.07E-01	galactose-1-phosphate uridylyltransferase
GALT	ARZ_4842_T1	1.3	4.37E-04	1.1	4.74E-01	1.2	4.30E-01	1.4	1.17E-01	1.6	1.47E-02	1.3	7.66E-03	galactose-1-phosphate uridylyltransferase
PGM	ARZ_3937_T1	1.1	5.29E-01	1.0	1.00E+00	1.3	7.84E-02	1.2	1.78E-01	1.4	6.49E-03	1.0	7.64E-01	phosphoglucomutase
PGM	ARZ_8190_T1	1.1	5.29E-01	1.1	8.80E-01	1.4	2.22E-05	1.2	3.24E-02	1.4	4.77E-06	0.9	2.51E-01	phosphoglucomutase
ME	ARZ_9592_T1	3.6	1.70E-59	1.6	4.20E-08	1.1	5.10E-01	1.5	2.70E-06	1.3	1.70E-03	0.7	1.30E-03	malic enzyme (NADP+)
ME	ARZ_1224_T1	3.5	2.50E-30	1.6	3.00E-04	1.2	4.00E-01	1.6	1.00E-02	1.4	4.70E-02	0.5	4.20E-08	malic enzyme (NADP+)

ME	ARZ_14261_T1	0.8	5.70E-01	1.6	1.30E-01	2.5	1.20E-19	2.0	2.00E-11	1.6	7.50E-06	1.2	1.10E-01	malic enzyme (NADP+)
ME	ARZ_13921_T1	0.8	2.60E-01	1.1	6.90E-01	1.9	1.10E-02	1.6	5.60E-02	1.7	2.70E-02	1.1	6.60E-01	malic enzyme (NADP+)
ME	ARZ_18809_T1	4.9	7.80E-114	2.0	2.90E-16	1.8	2.90E-08	1.9	6.20E-11	1.6	1.30E-05	0.8	2.20E-01	malic enzyme (NADP+)
ME	ARZ_1686_T1	1.0	1.00E+00	0.9	9.10E-01	1.3	4.50E-02	1.0	9.90E-01	0.8	1.70E-01	0.7	1.80E-02	malic enzyme (NAD+)
ME	ARZ_3656_T1	0.6	2.60E-09	0.8	2.80E-03	0.9	7.70E-01	0.7	1.40E-01	0.7	1.50E-01	0.7	1.50E-04	malic enzyme (NAD+)
