# Supplementary material for: Heterozygous diploid structure of Amorphotheca resinae ZN1 contributes efficient biodetoxification on solid pretreated corn stover
Source: Biotechnol Biofuels. 2019 May 21;12:126. doi: 10.1186/s13068-019-1466-z (PMC6528196; doi:10.1186/s13068-019-1466-z)
Supplement: Supplementary file 5 — Additional file 5: Figure S4. Comparison of the expression of orthologous gene pairs in central metabolism. [file 13068_2019_1466_MOESM5_ESM.docx]

**Figure S4** **Comparison of the expression of orthologous gene pairs in central metabolism.** Differentially expressed genes (fold change≥1.5 or ≤ 0.6, and FDR < 0.05) related orthologous gene pairs revolving with NAD(P)H cofactors production, ATP production or consumption, during the degradation of furfural (a), HMF (b), 4-hydroxybenzaldehyde (4-HBA) (c), vanillin (d), syringaldehyde (e), acetic acid (AA) (f), and formic acid (FA) (g). Abbreviations as the following, ATP-citrate synthase (ACLY), dihydroxyacetone kinase (DHAK), glucose-6-phosphate 1-dehydrogenase (GPDH), glucose transporter/sensor (2.A.1.1.68), glycerol kinase (GLK), glyceraldehyde 3-phosphate dehydrogenase (GAPDH), glycerol-3-phosphate dehydrogenase (GPD), glycerol 3-phosphatase (GPP), hexokinase (HK), high-affinity glucose transporter (2.A.1.1.39), isocitrate dehydrogenase (IDH), malate dehydrogenase (mitochondrial) (MDH1), malic enzyme (ME), monosaccharide transporter (2.A.1.1.57), monosaccharide transporter (2.A.1.1.58), α-oxoglutarate dehydrogenase (OGDH), 6-phosphofructokinase (PFK), 6-phosphogluconate dehydrogenase (PGD), pyruvate carboxylase (PC), pyruvate dehydrogenase (PDH), pyruvate kinase (PYK), ribose 5-phosphate isomerase (RPI), succinate dehydrogenase (SDH), succinyl-CoA synthetase (SCS), sugar/H^+^ symporter (2.A.1.1.69), transketolase (TK), and uncharacterized transporter (2.A.1.2.46).
