# Supplementary material for: Heterozygous diploid structure of Amorphotheca resinae ZN1 contributes efficient biodetoxification on solid pretreated corn stover
Source: Biotechnol Biofuels. 2019 May 21;12:126. doi: 10.1186/s13068-019-1466-z (PMC6528196; doi:10.1186/s13068-019-1466-z)
Supplement: Supplementary file 6 — Additional file 6: Figure S5. Gene ontology enrichment analysis of differentially expressed genes. [file 13068_2019_1466_MOESM6_ESM.docx]

**Figure S5** Gene ontology enrichment analysis of differentially expressed genes. GO terms of biological process (level 3) were analyzed and the significantly enriched catalogues (P-value ≤0.005) were presented. (a) Furfural. (b) HMF. (c) 4-Hydroxybenzaldehyde. (d) Vanillin. (e) Syringaldehyde. (f) Acetic acid. (g) Formic acid.
