# Supplementary material for: Heterozygous diploid structure of Amorphotheca resinae ZN1 contributes efficient biodetoxification on solid pretreated corn stover
Source: Biotechnol Biofuels. 2019 May 21;12:126. doi: 10.1186/s13068-019-1466-z (PMC6528196; doi:10.1186/s13068-019-1466-z)
Supplement: Supplementary file 7 — Additional file 7: Table S1. The primers used in this study. [file 13068_2019_1466_MOESM7_ESM.docx]

| Table S1 The primers for the validation of RNA-Seq data by qRT-PCR. | | |
| --- | --- | --- |
| Gene | Forward primer (5' to 3') | Reverse primer (5' to 3') |
| (a) qRT-PCR for RNA-Seq data under the degradation of furan aldehydes | | |
| ARZ_1967_T1 | CACGTCGTTGAAGCGAGAGTC | TTCTCATCCACTGCCTTGTAAGC |
| ARZ_7663_T1 | CAGGCCAGCACGTTGCTATAC | CAAGTCGTGTAGCTTCAGCGAG |
| ARZ_8436_T1 | TCAACATCACGTCTGGCTTGG | CTCTCTCTCGATCCGATCATTCTC |
| ARZ_11045_T1 | TCCACCGTTTCCCTGCCTAC | GGAGTTGGTAGAAGGAGTGCTGG |
| ARZ_12437_T1 | AAGCTGAAGGGAAGGGCAAG | ATCAAGCGCCAACCGAGTC |
| ARZ_12961_T1 | CCAGCAGACAAGAAGAGTCCTAAG | CACAACCTCCATCATTCGCTC |
| ARZ_13395_T1 | GGGACATTGTGCAATGGTGC | AGAGGCACGAAACCCTTCTGTAG |
| ARZ_15233_T1 | CCCGTCTCCTCTCCAAGTACC | AACATCCTCTTGCCAGTTGACC |
| ARZ_15318_T1 | ACAACCCTGATGAGGGCAAG | TGACAGCGAACTTGTGCTTAGC |
| ARZ_16609_T1 | AACTACATCGCTGGTTTTGGATC | CGTCAAAGAGCTGTTGGGAGA |
| ARZ_16710_T1 | CGGGAAGTATATCCAGACATACG | CTTCTTCCACATCTGGTGGATC |
| ARZ_16761_T1 | CATTCCAGATCCGACCCTCG | TTCCGCATCTCCTCCCTACTG |
| ARZ_16911_T1 | AACGAAAGTTGTCCTCCACTGG | AACGGTAATAGCAGCCTTCTCAAC |
| ARZ_16945_T1 | AAGGAGGGTCCAAGAGACCAG | AGTCTAAACGCAACTCTGAACACC |
| ARZ_17920_T1 | AGTGGACCAAGGATAGCATGAC | GACTAGAGCCTCGACGGTCTTC |
| ARZ_18111_T1 | CACGGTAGCAGACCTGTTTGAG | TGTGGTGAAGGAATTGATGAACC |
| ARZ_18349_T1 | CTTCCCATGCCTTCGTCATTAC | GTATCGGACCCTGTAACTTTGACC |
| (b) qRT-PCR for RNA-Seq data under the degradation of phenolic aldehydes | | |
| ARZ_99_T1 | GCTATCATGAACGGTCTCGCTG | GAACGTGAGACAGGGCAGACAG |
| ARZ_771_T1 | AAGGCTCTTGCCTTGCAGAGAT | TCATCCACCACAATCATGGGAT |
| ARZ_1621_T1 | TGATCATCGACCTGCTCCGATA | CTTCCGCAAGCTTCAGGAGAGT |
| ARZ_4254_T1 | CCCGAGTCTACGAGCGAATTAA | TCCCACAAAACATCAACCTTCG |
| ARZ_4835_T1 | CCCCAGGGACACGATAGCTCTA | GGTAGCTTGGCAAGGGGATCTT |
| ARZ_6146_T1 | TACGGAGGGAACCCTGTACTGG | ACCAGAAGCAACACGGAGGAAG |
| ARZ_9246_T1 | GCCCGAGGACTTCTGCTACAAG | ATCTCGGCCTGCTTGACGATGT |
| ARZ_9744_T1 | GCGATGGAGCGACCTATTTCTT | GCCATATTGACCGATGATGACG |
| ARZ_11538_T1 | TTGCTTGTCCTCCCTCAAGGAG | ATCCTCGAGGAACTGCTCCTTG |
| ARZ_13470_T1 | TGGACCAGAAACTGAGCGAAGA | AACATTCAGGGGCTTCAAAACG |
| ARZ_13958_T1 | CACCACATTCCTCATGTCGAC | GCCGTTCTTGTAGCAGAGCATG |
| ARZ_15588_T1 | AGTTGGAGACAGCTCTCCCAGC | TTGAAAGCCAAGTGGACTTCCC |
| ARZ_18006_T1 | ATCCCAAAATTCGTCAAGGAGG | GCCATCTTTGATATCAGCGGAT |
